# Confinement Effects in Catalysis Using Well-Defined Materials and Cages

**DOI:** 10.3389/fchem.2018.00623

**Published:** 2018-12-21

**Authors:** Valentinos Mouarrawis, Raoul Plessius, Jarl Ivar van der Vlugt, Joost N. H. Reek

**Affiliations:** Homogeneous, Supramolecular and Bio-Inspired Catalysis (HomKat) Group, Van 't Hoff Institute for Molecular Sciences (HIMS), University of Amsterdam, Amsterdam, Netherlands

**Keywords:** heterogeneous catalysis, homogeneous catalysis, zeolites, supramolecular chemistry, metal-coordination cages

## Abstract

This review focuses on the effects that confinement of molecular and heterogeneous catalysts with well-defined structure has on the selectivity and activity of these systems. A general introduction about catalysis and how the working principles of enzymes can be used as a source of inspiration for the preparation of catalysts with enhanced performance is provided. Subsequently, relevant studies demonstrate the importance of second coordination sphere effects in a broad sense (in homogeneous and heterogeneous catalysis). Firstly, we discuss examples involving zeolites, MOFs and COFs as heterogeneous catalysts with well-defined structures where confinement influences catalytic performance. Then, specific cases of homogeneous catalysts where non-covalent interactions determine the selectivity and activity are treated in detail. This includes examples based on cyclodextrins, calix[n]arenes, cucurbit[n]urils, and self-assembled container molecules. Throughout the review, the impact of confined spaces is emphasized and put into context, in order to get a better understanding of the effects of confinement on catalyst performance. In addition, this analysis intends to showcase the similarities between homogeneous and heterogeneous catalysts, which may aid the development of novel strategies.

## Introduction

Catalysis occupies a pivotal role in the modernization of our chemical industry, because it ensures more efficient use of natural resources and also aids in the minimization of waste production. For these reasons, the development of efficient catalytic processes is of crucial importance for sustainable-oriented applications, which in turn positions catalysis at the heart of our quality of life (Beller, 2011). Catalytic performance can be described by three key parameters, activity, selectivity, and stability. The activity of a certain catalyst is dictated by the rate determining step of the catalytic cycle. Obtaining detailed insight in the rate determining step can be difficult and time consuming, which limits the development of novel and highly effective catalyst systems by rational design. Whereas, homogeneous catalysts consist often of a single active species, heterogeneous catalysts mostly contain different metal sites in mechanically stable (porous) materials with high surface area (Bell, 2003).

In other words, there is a diverse range of active sites present where elementary steps can occur, each potentially with different catalytic activity. This increases the complexity of a particular catalytic system and makes the prediction of the performance of a new catalytic material challenging (Honkala et al., 2005). For homogeneous (transition) metal catalysts it is more facile to obtain molecular-scale information of the active species, which in turn aids in unraveling the reaction mechanism of specific reactions (Cornils and Herrmann, 2002; Ferreira et al., 2004). It is now well-recognized that the use of electronically and/or sterically tuned ligands can lead to enhanced catalytic performance of the corresponding metal complexes (Trost et al., 1992; van Leeuwen, 2004; Praneeth et al., 2012; Farkas et al., 2013; Karroumi et al., 2015). Nonetheless, the rational design of tailor-made catalysts for specific transformations is still in its infancy, and systematic screening of catalysts is the main practice to “discover” new catalysts. Hence, detailed insight in all aspects that determine the catalyst properties is required to allow the development of catalysts-by-design.

Enzymes, nature's catalysts, have served as a source of inspiration for scientists in the field of catalysis (Breslow, 1995; Dodziuk, 2006; Wang et al., 2013). These systems typically incorporate multiple functionalities inside their catalytically relevant cavity, in order to enforce high selectivities and activities. Even though the working principles of enzymes are still subject to debate, advances in transition state theory and computer simulations have allowed the formulation of general principles that can be translated into synthetic systems (Garcia-Viloca et al., 2004). An essential aspect in this context is the preservation of a well-defined confined space (second coordination sphere) around the active center, for a number of reasons. First, it ensures proximity of substrate(s) and the catalyst active site, thereby enhancing overall reaction rates by just pre-organization. Secondly, residues near the active site can affect substrate binding and as such substrate selectivity can be achieved (Steitz et al., 2008). Thirdly, enzymes can pre-organize the substrate in a higher energy conformation, which results in increased reactivity (Pompliano et al., 1990). Most importantly, according to transition state theory, binding of the transition state should be stronger than the substrate, leading to enhanced rates. Along the same lines, the cage can also destabilize intermediates to lower the transition state barriers (Alber et al., 1983). These effects describe in general terms how the second coordination sphere surrounding the active site of an enzyme contributes to enhanced catalyst performance. Inspired by these examples from Nature, there is a growing interest in concepts that enable the synthesis of well-defined second coordination spheres in order to prepare enzyme mimics (Raynal et al., 2014). Furthermore, the application of catalysis in confined spaces, in order to take advantage of the second coordination sphere effects, has received increasing attention (Vriezema et al., 2005). One of the first reports of a confined space effect in catalysis by a synthetic compound involved the use of cyclodextrins (Crini, 2014). Thereafter, other important classes of “confined” host molecules have been developed, including metal-organic cages, metal-organic frameworks, cyclodextrins, cucurbit[n]urils, and nanoreactors. Also in the area of heterogeneous catalysis the role of the second coordination sphere is widely recognized, and many types of porous materials are currently used. This review aims to illustrate the influence of confinement on the selectivity and activity in catalysis, by highlighting relevant studies in both heterogeneous and homogeneous catalysis. We outline the impact of well-defined cavities on catalytic activity and selectivity. This includes zeolites, metal-organic frameworks, covalent-organic frameworks as well as cyclodextrins, calix[n]arenes, cucurbit[n]urils, and self-assembled container molecules. Relevant studies will be put into context with the aim to provide fundamental understandings on how confinement effects can influence selectivity and activity in catalysis.

## Confinement Effects in Heterogeneous Catalysis

The so-called confinement effects in porous materials are known to strongly affect diffusion, phase transformations and as such the catalytic properties when applied as catalyst material. The resulting confined environment can result in changes in specific reactions due to adsorption, geometrical constraints, selective absorption, and changes to the potential energy surface that can eventually influence the reaction in terms of selectivity and activity. Herein we highlight recent examples that illustrate the influence of confinement effects, which in turn govern the activity and selectivity in heterogeneous catalysis (zeolites, MOFs, and COFs).

### Zeolites

**Figure 1:**
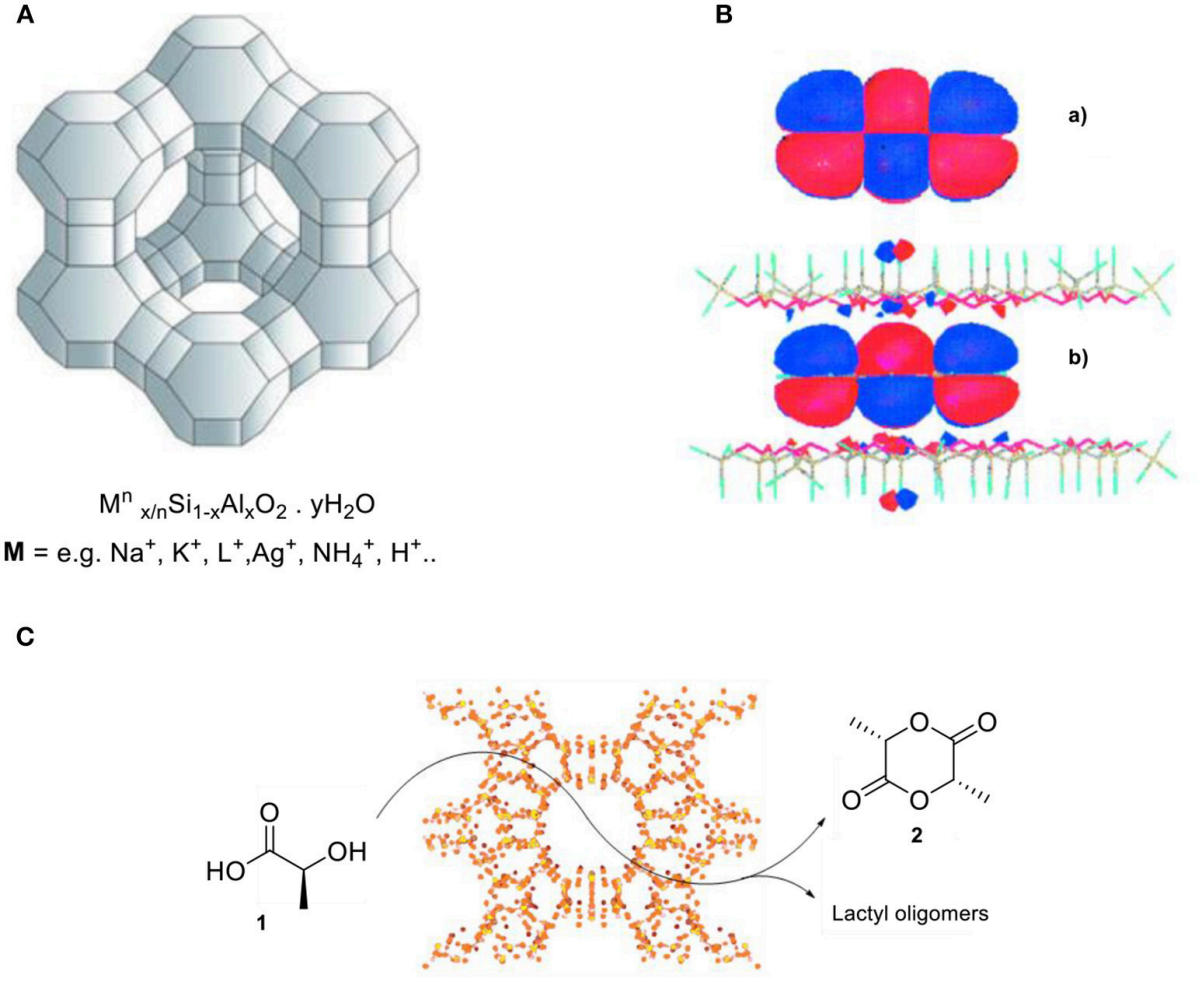
**(A)** Representation of zeolite structure and its general chemical composition. **(B)** Calculated spatial extension of the HOMO of anthracene **(a)** in the gas phase and **(b)** when it is confined between two sheets of silicate. Reproduced with permission from Márquez et al. (2001). **(C)** An alternative route for lactide formation by utilizing zeolite H-Beta catalyst.

Zeolites are a class of crystalline, microporous silicates, or aluminosilicates, that form well-defined pores and cavities of molecular dimensions (ca. 3–12 Å) within their structure (Figure 1A; Cundy and Cox, 2005). The application of zeolites led to the replacement of high-value processes that were based on mineral acids as stoichiometric and often toxic reactants, as it enabled the development of more environmentally friendly and resource-efficient processes with lower byproduct formation and energy consumption as essential advantages (Dusselier and Davis, 2018). Zeolites are the most widely used class of heterogeneous catalysts, with applications for these inorganic materials in over one hundred industrial processes involving catalysis, adsorption/separation, and ion exchange (Cejka et al., 2010). The broad applicability range is primarily due to their highly tunable chemical composition and high degree of porosity. This results in non-covalent interactions (Pauli repulsion and van der Waals type of interactions) between the zeolite framework and molecules within their confined free space (Derouane, 1997). A comprehensive study reported by the group of Garcia suggests that molecules in zeolites are confined at the molecular level (Figure 1B). In other words, the molecular orbitals are strongly influenced by the solid material. For example, the aromaticity of anthracene in zeolites is strongly disrupted due to the limitation of the *p*-orbital spatial extension induced by the pore walls' proximity (Márquez et al., 2001).

One of the first studies demonstrating the effect of shape-selective reactions by zeolites related to the selective alkylation of toluene with methanol to produce a mixture of xylene isomers as the major products, by suppressing the formation of other isomers that can be formed under free movement conditions (Kaeding et al., 1981). It has been rationalized by the steric hindrance for alkylation within the confines of the catalyst pores. Further modification of the zeolite by controlled calcination in the presence of phosphorus sources (e.g., H_3_PO_4_) resulted in a dramatic increase in the *para*-isomer of xylene. The large positional selectivity was attributed to the shape selectivity of the particular zeolite with a more confined cylindrical pore. In addition, the bulky phosphorus atom partly blocks the pore openings of the zeolite, favoring the diffusion of the *para*-xylene over *meta*- and *ortho*-isomers. The porous structure of zeolites that are impregnated with metals unexpectedly leads to high selectivity for the formation of benzene in the aromatization of *n*-hexane (Kanai and Kawata, 1989; Ayad, 2002). This high selectivity to aromatics partially stems from the constrained environment set by the shape of the particular zeolite which enforces cyclization of the *n*-hexane molecule. This is a case of an effective substrate pre-organization within a confined space.

Selective catalytic hydrogenations are still highly valuable when multiple reducible groups are present in the reactant. The group of Feng-Shou Xiao developed a Pd@zeolite catalyst where high catalytic activity (metal nanoparticles) and selective adsorption of reactants (zeolite micropores) are combined in one material (Zhang et al., 2017b). This resulted in higher selectivity for hydrogenation of the nitro group of nitroarenes in comparison with commercial Pd/C catalyst and Pd nanoparticles supported on zeolite crystals. The superior selectivity was attributed to the selective adsorption of the nitroarenes on the Pd nanoparticles forced by the zeolite micropores.

Selective formation of lactide has been reported by Sels and co-workers using a shape-selective zeolite (Dusselier et al., 2015; De Clercq et al., 2018). They developed a zeolite-based catalytic process that converts lactic acid **(1)** into lactide **(2)** by utilizing the Brønsted acidic H-Beta zeolite, which is able to suppress the formation of unwanted oligomers. The use of other common catalysts such as sulfuric acid or other Brønsted acidic zeolites with bigger pore diameters than H-Beta resulted in lower selectivity to lactide, because of competing lactic acid condensation to lactyl oligomers (Figure 1C).

Zeolites can be hydrophilic or hydrophobic depending on the charges present. In particular, the selectivity of zeolites as adsorbents for polar or non-polar molecules can be tuned in several ways. For example, the addition of an extra framework of cations in the zeolite structure or a change in the Si/Al ratio can result in major changes in the polarity of the surface of the material, and thus the confined space.

The tunable character of these materials adds an extra tool to control the activity and selectivity of the catalyst. When reactants with unequal polarities are used, these will be absorbed in the zeolite pores in different ratios, and as such one can generate a substrate-selective material (Ogawa et al., 1994; Climent et al., 2000). This is particularly important in the oxidation of organic compounds using aqueous H_2_O_2_. An illustrative study is the olefin epoxidation reaction with a titanium-based zeolite catalyst for the formation of the corresponding epoxide. By lowering the polarity of the cavity the epoxide selectivity increased from 26% to over 96% (Corma, 2003).

### Metal-Organic Frameworks (MOFs)

**Figure 2:**
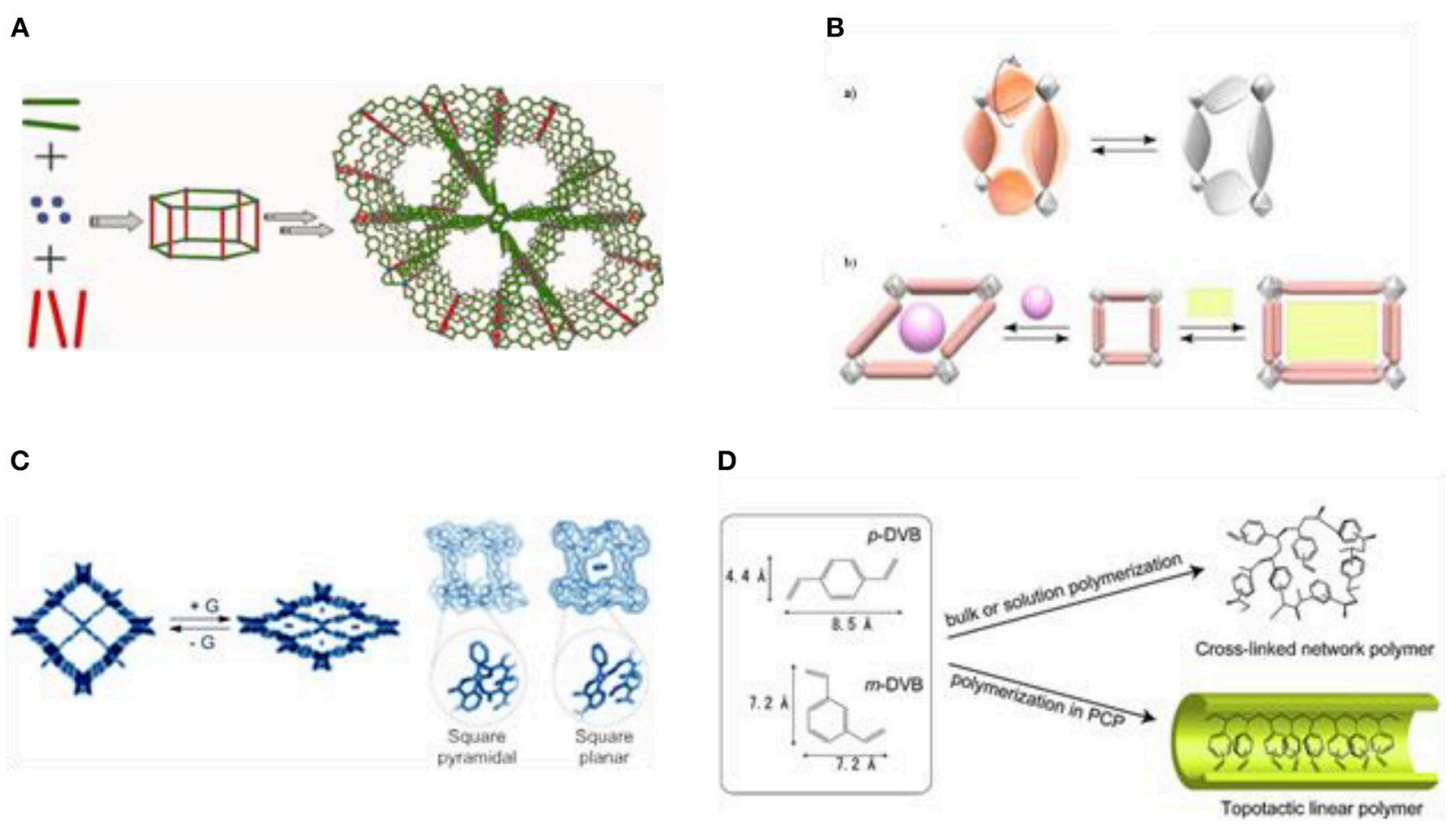
**(A)** A common generalized scheme of MOF synthesis. **(B,a)** Schematic representation of dynamic structure property of MOFs, **(a)** rotation of bridging ligands and **(b)** shape-responsive fitting. **(C)** A shape responsive MOF system, where G = benzene. **(D)** Bulk polymerization vs. polymerization in PCP (pillared [M_2_(bdc)_2_(teda)n]). Reproduced with permission from Uemura et al. (2007).

Metal-organic frameworks (MOFs) present an important class of novel materials that allow the generation of well-defined structured materials using molecular building blocks (Batten et al., 1995; Ahmad et al., 2014). These solids exhibit attractive features, most importantly their crystalline nature, permanent porosity, high specific surface area (Farha et al., 2012), large pore aperture (Deng et al., 2012), and low density (Furukawa et al., 2011). They are typically composed of two key components, i.e., inorganic vertices (metal ions or clusters) and organic linkers. The application of well-known coordination chemistry together with the utilization of organic building blocks with tailor-made structures result in the formation of networks with defined topologies, depending on the structures of the respective building blocks and the relative ratios used (Figure 2A). Comparing MOFs to zeolites reveals many differences that can significantly influence catalysis in terms of selectivity and activity. For example, although the number of known zeolite structures is substantial, the molecular diversity of the building blocks should allow for an almost infinite variety of MOFs. MOFs benefit from the versatile coordination chemistry, polytopic linkers, and terminating ligands that are employed. This difference to zeolites is nicely illustrated in the self-assembly of the zeolitic imidazolate frameworks (ZIFs), where a slight change in the ligand composition results in a dissimilar network and pore structure (Banerjee et al., 2008). It is known that the small pore size of zeolites can be a limiting factor in catalytic reactions that involve large molecules. Hence, intensive research has been devoted to the development of zeolites with very large pores (Paillaud et al., 2004; Corma et al., 2006). On the contrary, many MOFs with a variety of pores have already been reported (ultramicroporous-mesoporous). MOFs are also known to exhibit dynamic features and they can be guest-responsive (Figure 2B). This property often appears in MOFs that contain organic parts that can undergo rotation under the influence of radiation or heat (Lee et al., 2005). The pillared layer compound CPL-2 exhibits shape-responsiveness when benzene is used as guest molecule. Benzene structurally influences this particular MOF as the Cu coordination changes from square pyramidal to square planar (Figure 2C) (Matsuda et al., 2004; Kubota et al., 2006).

This feature has been applied in the polymerization of acetylene using a porous coordination polymer [Cu_2_(pzdc)_2_(bpy)], where pzdc is pyrazine-2,3-dicarboxylate and pyz is pyrazine and exploiting a molecular recognition mechanism that is accompanied by structural transformation (Matsuda et al., 2005). The MOF resulted in a dramatic acceleration of the polymerization reaction, without production of tri-substituted benzenes as by-products, and proceeded in a *trans*-selective fashion. The authors attributed the *trans*-selectivity to the constrained environment present in the nanochannels of the MOF. This steric phenomenon enables strong interactions between the monomer and carboxylate groups as it forces the two reactants to be arranged at proper positions (Uemura et al., 2006). The last feature of MOFs is their ability to perform bimolecular reactions between two reactants of very different polarities. The catalytic efficiency of a particular MOF can be altered by regulating the hydrophobicity of the porous solid.

Efficient catalytic polymerization processes are still highly desirable. Important steps forward have been taken in the development of MOF-based polymerization processes. Interestingly, leading chemical companies have invested a lot in the research and development of such processes (Müller et al., 2003). A study revealed that the radical polymerization of divinylbenzene in the pillared [M_2_(bdc)_2_(ted)n] (M = Zn^2+^, Cu^2+^, bdc = benzenedicarboxy-late, ted = triethylenediamine) can be carried out with the use of a radical precursor (2,2′-azobisisobutyronitrile). In particular, a topotactic selective radical polymerization has been successfully achieved inside the one-dimensional nanochannels of the applied MOF. The authors suggested that the size of the pores and the flexibility of the framework are the key features of the MOF structure, responsible for the high selectivity (Figure 2D). In correlation with the rationale for this high selectivity, the use of MOFs with larger pores resulted in non-selective polymerization with the formation of cross-linked network polymers (Uemura et al., 2007).

One of the benefits that the confined space of MOFs offers is the high surface area. This can lead to a high and monodisperse loading of catalytically active species, which in turn could result in better performance. Examples of nanosized metal particles loaded into the pores of MOF-5 and MOF-177 mostly include Pt, Au, Pd, and Ru (Hermes et al., 2005; Schröder et al., 2008). The Pd-supported MOF-5 catalyst has been evaluated in the catalytic hydrogenation of various alkenes and the activity of Pd/MOF-5 was twice as high as that of a commercial Pd/C catalyst (Opelt et al., 2008). A different study showed that the use of the Au/MOF-5 catalyst can lead to enhanced activity in the aerobic oxidation of benzyl alcohol in methanol (Ishida et al., 2008). Klemm and coworkers reported a different MOF system, [Pd(2-pymo)_2_]_*n*_, (2-pymo = 2-pyrimidinolate) for the hydrogenation of 1-octene and cyclododecene (Opelt et al., 2012). The confinement effects resulted in shape- and size-selectivity, because 1-octene was hydrogenated but not the more bulky cyclododecene.

MOFs with Brønsted-basic properties can combine catalytic reactivity with the advantages of the confined environment, for example by incorporating basic functional groups such as amines and pyridines. Hartmann and coworkers published a synthetic protocol for the synthesis of three different basic MOFs: NH_2_-MIL-101(Fe), NH_2_-MIL-101(Al), and CAU-1 (Hartmann and Fischer, 2012). In each of them the presence of non-coordinated primary amines leads to the ability to perform Brønsted-basic catalysis, even without the assistance of polar or protic solvents. The new functionalized MOFs were evaluated for the base-catalyzed Knoevenagel condensation of benzaldehyde with ethyl cyanoacetate. The amino-functionalized MOFs NH_2_-MIL-101(Fe) and NH_2_-MIL-101(Al) proved excellent catalysts for the formation of the Knoevenagel condensation products (≈90% yield) and significantly more active than classical solid bases such as MgO and hydrotalcite. On the other hand, CAU-1 exhibited a poor activity, which has been attributed to transport limitations due to the small windows of the structure.

MOFs can also be multifunctional, a feature that further expands the potential of these materials, for instance in specific applications that need a catalyst that combines the influence of a confined space with an acid and/or a base character. The group of Duan reported a new approach to construct a multifunctional lanthanide-organic framework (Tb-TCA, where H_3_TCA = tricarboxytriphenylamine) that features a high concentration of Lewis-acidic Tb^3+^ sites and Lewis-basic triphenylamine sites on its internal surfaces. Tb-TCA shows high activity in the Knoevenagel condensation of salicylaldehyde derivatives with malononitrile and cyanosilylation of different benzaldehydes and cyanotrimethylsilane in a size-selective way through basic and acidic catalytic sites, respectively (Wu et al., 2011).

**Figure 3:**
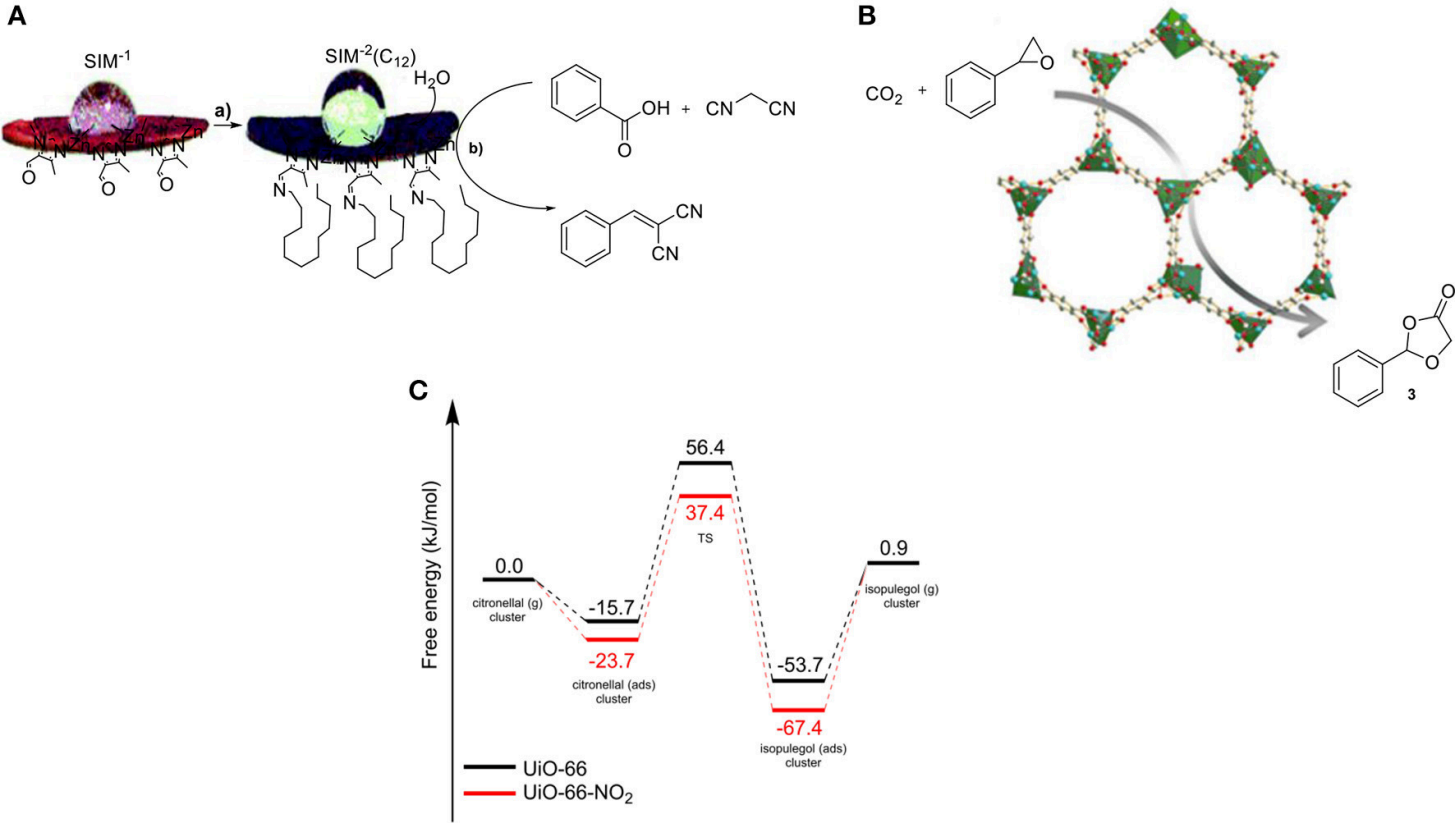
**(A,a)** Synthesis of SIM-2(C12) and **(b)** Knoevenagel condensation. **(B)** Illustration of the cycloaddition of CO_2_ and epoxides by utilizing a MOF. **(C)** Gibbs free-energy profiles for citronellal cyclization by utilizing the NO_2_-functionalized and non-functionalized MOF.

Poisoning by water is a common problem in the field of catalysis and the discovery of methods that can exclude water throughout a reaction is highly valuable. Water can originate from moisture in the air or it can be formed during the reaction. MOFs with long alkyl chains within the MOF structure can prevent the penetration of water. The group of Zhong introduced a methyl group onto different positions of the bipyridine-pillar linker in the particular MOF, suppressing the adsorption of water molecules on the catalytic sites (Ma et al., 2011). Several studies demonstrating this effect in MOF catalysis have been reported by the group of Farrusseng (Canivet et al., 2011). They developed a new hydrophobic MOF by post-synthetic modification of SIM-1 and used it as a catalyst in Knoevenagel condensation. The modified catalyst SIM-2(C_12_) showed higher catalytic activity in the Knoevenagel condensation compared to SIM-1 due to the creation of a hydrophobic environment surrounding the catalytic sites. Specifically, SIM-2(C_12_) was 3-fold more efficient than SIM-1 (Figure 3A). In another report, cycloaddition of CO_2_ to styrene epoxide has been investigated. The use of a MOF (Co-MOF-74) containing Lewis-acid sites resulted in high catalytic activity in addition to a high yield of 96% to the corresponding product (**3**, Figure 3B) (Cho et al., 2012). In this perspective, MOFs can be utilized for the efficient CO_2_ adsorption, followed by a catalytic reaction within their cavities. However, further investigations are required in order to develop a proficient and large-scale process that uses gaseous CO_2_.

The effects of modification (electronic and steric) of the cavity walls on the performance of a catalyst enables in-depth studies on the effect of the side-walls in catalysis. The group of Van Speybroeck and De Vos investigated the effect of substituents on the linkers of a MOF (Vermoortele et al., 2012). The cyclization of citronellal was chosen to uncover the catalytic performance of these electronically modified MOFs. The results showed that the rate of the reaction was significantly enhanced by installing electron-withdrawing groups on the organic building block, with the nitro-group providing the most active material. A linear free-energy relationship (Hammett-type LFER) was found between the degree of electron-withdrawing character of the substituent on the linker and the reaction rate in the carbonyl-ene reaction. That was the first LFER ever observed in MOF catalysis. The authors have been able to use molecular modeling to get insight into the energy levels for the reactant, transition state, and product for both the non-substituted and the NO_2_-substituted MOF (Figure 3C). The substitution alters the Lewis-acidic properties and most importantly induces additional stabilizing or destabilizing effects on the reactants, depending on whether the substituent is electron-withdrawing or -donating, respectively. In case of a NO_2_ group as a substituent on the MOF linker, the absorption and activation energy of the reaction are lowered but also a more stabilized transition state is generated.

**Figure 4:**
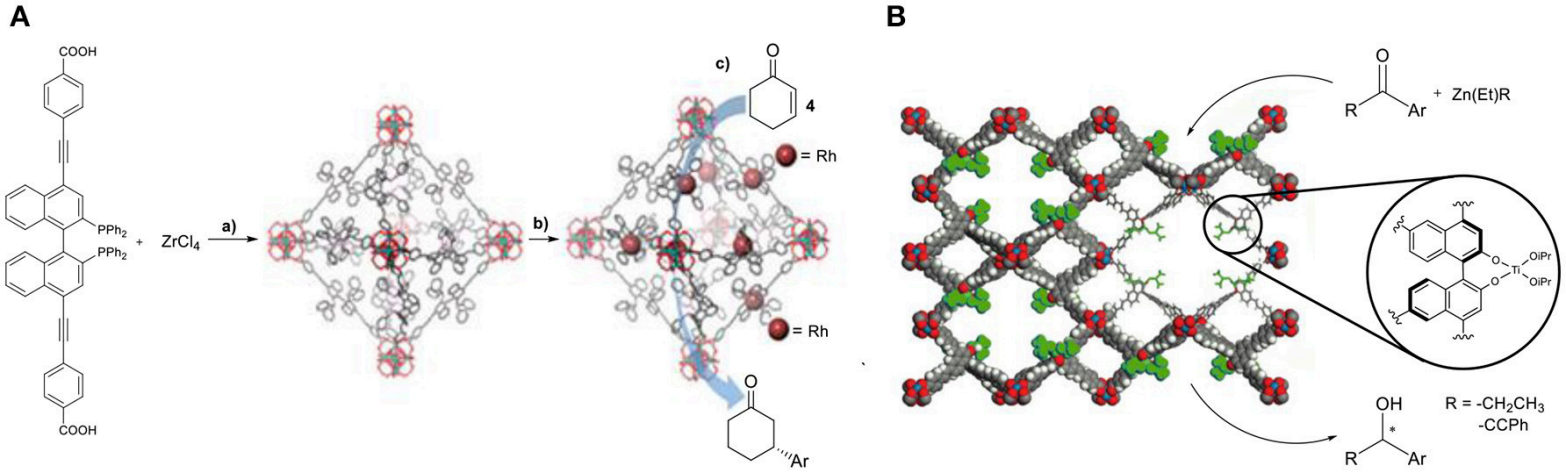
**(A,a)** Synthesis of the chiral Zr-MOF, **(b)** post-synthetic metalation **(c)** asymmetric addition of aryl-boronic acid. Reproduced with permission from Falkowski et al. (2014). **(B)** Asymmetric alkynylzinc additions catalyzed by the chiral MOF/Ti-BINOLate catalyst. Image adjusted from Ma et al. (2010).

The area of heterogeneous asymmetric catalysis is a developing field. MOFs can serve as tunable platforms for the development of efficient asymmetric catalysts. In this direction, several well-defined ligands have been used to construct MOFs aiming for applications in asymmetric catalysis. In particular, a rhodium post–synthetic functionalized MOF was an exceptional catalyst in the conjugated addition of arylboronic acid to cyclohex-2-en-1-one (**4**, Figure 4A) (Falkowski et al., 2014), displaying excellent enantioselectivities of up to 99% and three times higher activity than the homogeneous control reaction.

Other examples of privileged chiral ligands that have been incorporated in the synthesis of MOFs able to induce chirality are based on dicarboxylate-functionalized Ni(salen) and Co(salen)(OAc). Ma and coworkers used eight different chiral tetracarboxylate ligands in the synthesis of mesoporous chiral MOFs (Figure 4B). These systems showed high activities for diethylzinc and alkynylzinc additions to aromatic aldehydes. It has been well-demonstrated in these systems that enantioselectivities correlate strongly to the size of the cavities (Ma et al., 2010).

An interesting application of MOFs as catalytic materials is the inclusion of biomimetic active sites, such as metalloporphyrins. The thermally and chemically stable MOF frameworks have the potential to stabilize the metalloporphyrin, create reactive pockets and enhance diffusion. For example, the Zr-MOF prepared by the group of Zhou proved to be very stable, with a high density of metalloporphyrinic sites and an excellent peroxidase-like catalytic activity. The latter was attributed to the large open channels that allow efficient porphyrin encapsulation, yet efficiently prohibit the self- dimerization of porphyrin centers (Feng et al., 2012).

### Covalent-Organic Frameworks (COFs)

**Figure 5:**
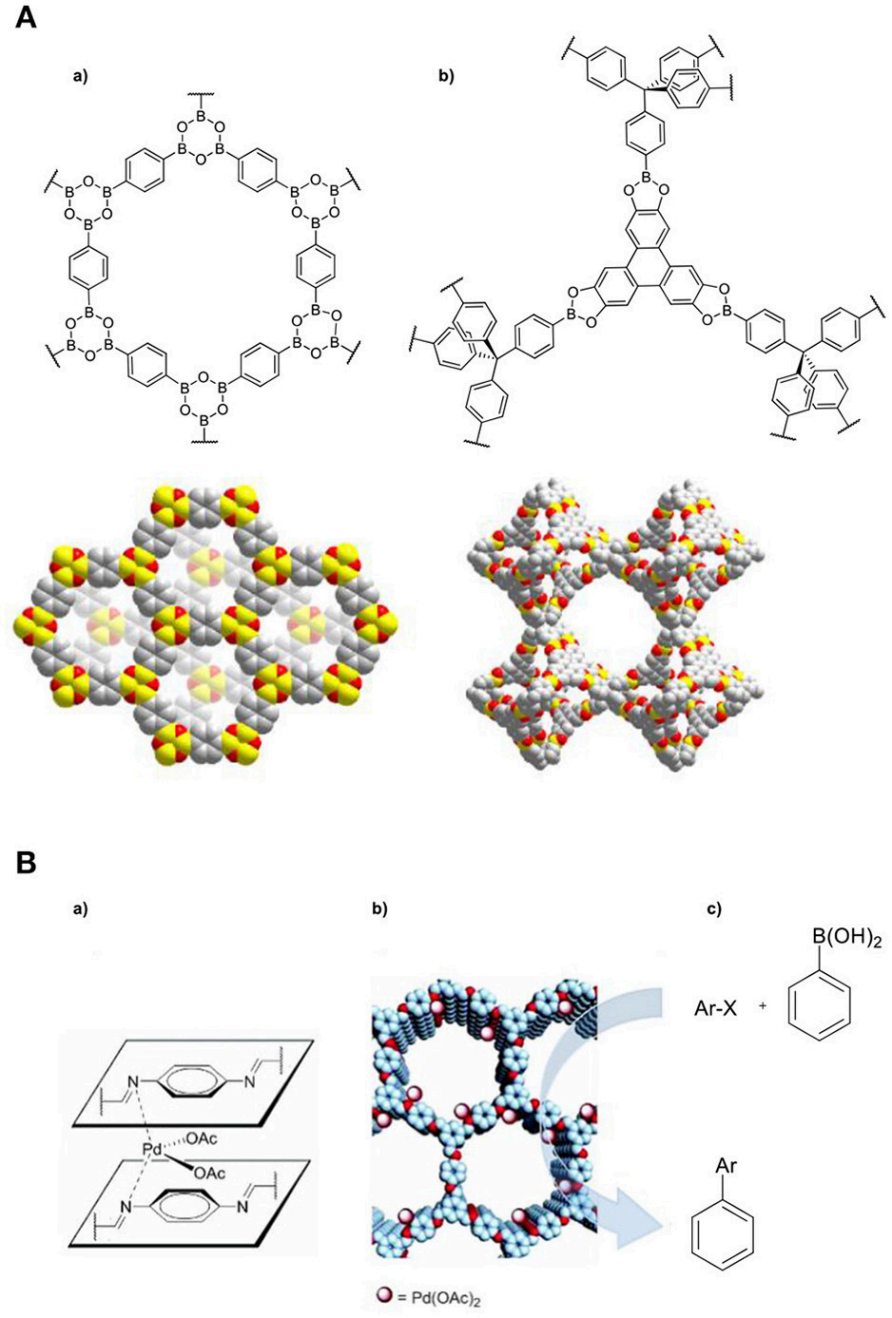
**(A)** Structure of a fragment and space-filling diagram of the powder X-ray crystal structure of **(a)** COF-1 and **(b)** COF-108. Reproduced with permission from Waller et al. (2015). **(B)** Illustration of **(a)** the confined (Pd(OAc)_2_ catalyst **(b)** proposed structure of Pd/COF-LZU1 and **(c)** Suzuki-Miyaura coupling reaction. Reproduced with permission from Ding et al. (2011).

COFs are relatively new class of materials and related to MOFs. The difference is that COFs are constructed by the formation of (reversible) covalent bonds, rather than coordination chemistry, resulting in crystalline porous organic polymers (Figure 5A) (Côté et al., 2005; Waller et al., 2015). The structure and the geometry of these organic materials can be fine-tuned by the directionality of the covalent bonds used, which in turn can lead to the rational design of these materials. In general COFs have rigid structures, excellent thermal stability, and they exhibit permanent porosity (Diercks and Yaghi, 2017). For example, COF-1 consists of extended layers stacked in staggered form to give hexagonal pores of 15 Å diameter and a BET surface area of 711 m^2^ g^−1^.^64^ COF-108 (El-Kaderi et al., 2007). has three-dimensional pores and an extremely low density. A recent study by the group of Yushan reported the synthesis and the catalytic application of two 3D microporous base-functionalized COFs (BF-COF-1 and BF-COF-2) (Fang et al., 2014). These materials have been used as catalysts in Knoevenagel condensation reactions with a variety of substrates where they showed high size-selectivity and good recyclability. The size-selectivity has been illustrated by employing different size substrates (benzaldehyde, 4-methylbenzaldehyde and malononitrile), where only the smallest substrate (benzaldehyde) was converted.

An alternative approach to generate catalytic active COFs is via post-synthetic modification to introduce metal sites into their framework. Wang and coworkers reported the incorporation of (Pd(OAc)_2_ into an imine-linked COF to generate Pd/COF-LZU1 (Ding et al., 2011), which has been used for the Suzuki-Miyaura coupling reaction (Figure 5B). It showed superior catalytic performance in terms of activity, as it required a lower catalyst-loading, shorter reaction time and displayed higher reaction yield in comparison to other crystalline porous materials (MOFs and zeolites). Recently, a series of chiral COFs proved to be efficient and recyclable heterogeneous catalytic platforms for several types of asymmetric organic reactions (Han et al., 2017; Zhang et al., 2017a).

## Confinement Effects in Homogeneous Catalysis

The encapsulation of a catalyst in a molecular container with a well-defined confined space imposes the so-called second coordination sphere effects, which can influence the activity and selectivity of the catalytic reaction. In this section, we highlight some important examples that demonstrate the influence of confinement effects set by different hosts (cyclodextrins, calixarenes, cucurbiturils, and self-assembled container molecules) that govern the activity and selectivity in homogeneous catalysis in confined spaces.

### Cyclodextrins (CDs)

**Figure 6:**
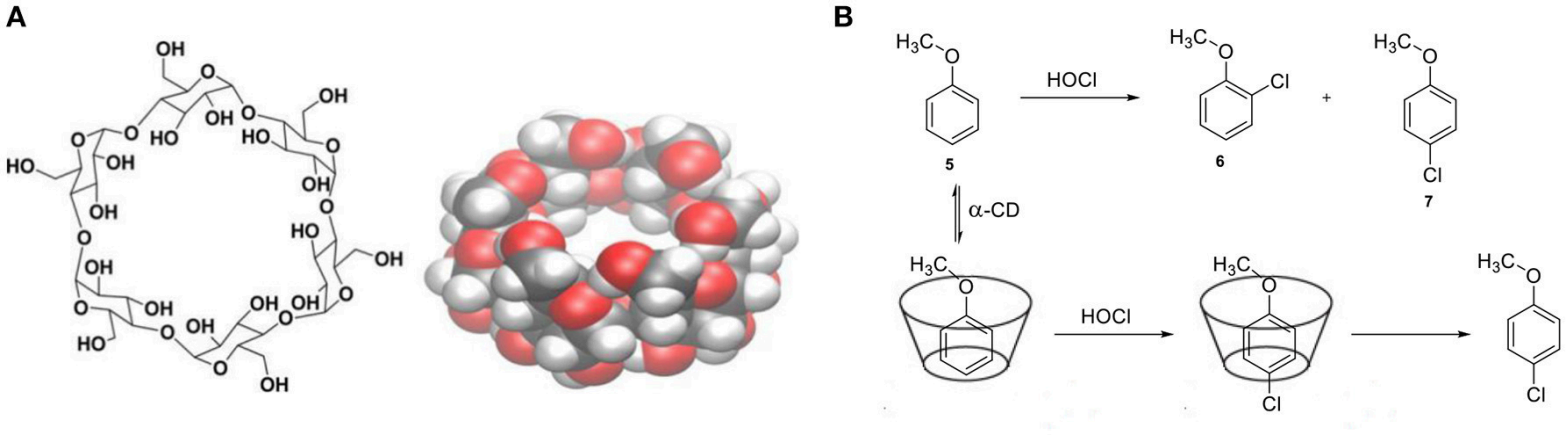
**(A)** Schematic and space-filling structures of α-cyclodextrin. **(B)** Chlorination of anisole (**5**) within the cavity of α-cyclodextrin.

**Figure 7:**
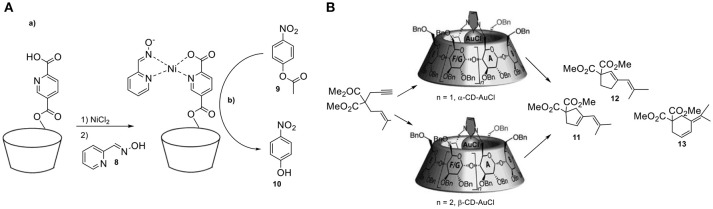
**(A,a)** The preparation of the supramolecular nickel catalyst and **(b)** hydrolysis of *para*-nitrophenylacetate (**8**). **(B)** Product-selectivity controlled by the size of the cyclodextrin.

**Figure 8:**
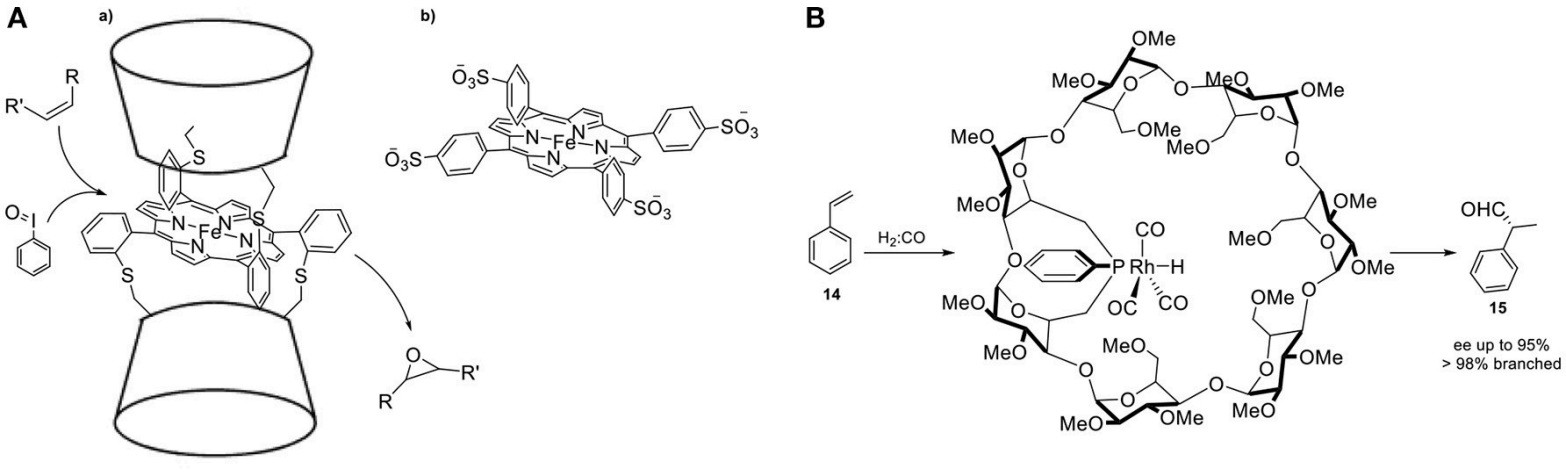
**(A,a)** β-CD sandwiched iron-porphyrin catalyst **(b)** and its analog deprived of cavity. **(B)** Confined monophosphine–rhodium catalyst in cyclodextrin for the asymmetric hydroformylation of styrene.

CDs have attracted much attention as molecular structures with well-defined cavities and they have been first applied as simple enzyme models where confinements effects appear to influence activity and selectivity in catalysis (Hapiot et al., 2011)_._ They are readily available cyclic oligomers of α-D-glucopyranoside monomers, soluble in water and with a defined, hydrophobic inner cavity. The most common are the α-, β-, and γ-CDs with a cavity diameter ranging between ≈5.6 and 8.8 Å. Their structure consists of two rims, both containing a network of hydroxyl groups (Figure 6A). One of the first examples illustrating the influence of confinement in the selectivity has been reported by the group of Breslow. They demonstrate that by encapsulating a substrate, control over the regioselectivity can be achieved (Figure 6B) (Breslow and Campbell, 1969). In particular, they reported the regioselective chlorination of anisole (**4**) with hypochlorous acid by performing the reaction in the cavity of a cyclodextrin. Importantly, chlorination of anisole (**5**) usually results in the formation of a mixture of *ortho*- (**6**) and *para*- chloroanisole (**7**). Hence, the environment of CDs shield the *ortho* sites from substitution due to the specific orientation in the cavity of the host. Breslow and Overman functionalized CDs with metal complexes to induce pre-organization around the active catalyst (Breslow and Overman, 1970). It has been suggested that attachment of a suitable hydrophobic host could increase the activity of a transition metal catalyst. The supramolecular catalyst has been prepared by covalent anchoring of pyridine-2,5-dicarboxylic acid to α-cyclodextrin, followed by treatment with NiCl_2_ and pyridine carboxaldoxime (**8**, Figure 7A). The catalytic performance of these confined catalysts has been demonstrated in the hydrolysis of *para*-nitrophenylacetate (**9**) to form nitrophenol (**10**) and it resulted in superior activity with respect to the free catalyst. More recently, Sollogoub and coworkers studied the effects of confinement on a gold-carbene catalyst encapsulated in the cavity of a cyclodextrin (Guitet et al., 2013). To show the influence of the microenvironment they studied an intramolecular cyclization reaction. The selectivity was controlled by the size of the cavities, because the smaller α-cyclodextrin gold-carbene catalyst led to both five-membered ring products (**11**, **12**), while the bigger β-CD resulted in the formation of mostly **13** (Figure 7B). The performance of the same catalyst in an asymmetric cycloisomerization version from enyne proved to be promising with enantioselectivities up to 59%. Kuroda and coworkers reported an iron-porphyrin complex sandwiched between two β-CD cavities (Figure 8A) (Kuroda et al., 1989, 1990). The activity of the unbound porphyrin, i.e., without entrapment by the CD moieties, was significantly lower with respect to the sandwiched catalytic system in the epoxidation of alkenes. More specifically, the yield for cyclohexene epoxidation by the confined system was 55% whereas in the absence of the cavity the yield dropped dramatically to < 2%. The authors propose that decomposition of the reactive Fe = O species outcompetes reaction with the substrate when the metal center is not shielded off by the cavity. Therefore, the presence of the cyclodextrin prevents the trapping of the reactive species by another iron complex, while keeping the access to the metal center open for substrate epoxidation. CDs present a suitable platform for control over the stereochemistry as they consist of enantiopure monomers. The group of Matt reported in 2014 the use of a monophosphine-rhodium complex appended to a α- or β-cyclodextrin, applied in the asymmetric hydroformylation of styrene (**14**) (Jouffroy et al., 2014). Specifically, due to the steric bulk present on the cavity- functionalized ligands, the confined complexes consist of a rhodium center exclusively bound to a single PR_3_ ligand. The performance of this catalyst in the asymmetric hydroformylation of styrene proved to be highly regioselective and enantioselective, affording **15** in 95% ee (Figure 8B). This stereocontrol can be attributed to the chiral cyclodextrin preferentially delivering the hydride and formyl fragments to one of the two enantiotopic faces of the alkene.

### Calix[n]arenes

**Figure 9:**
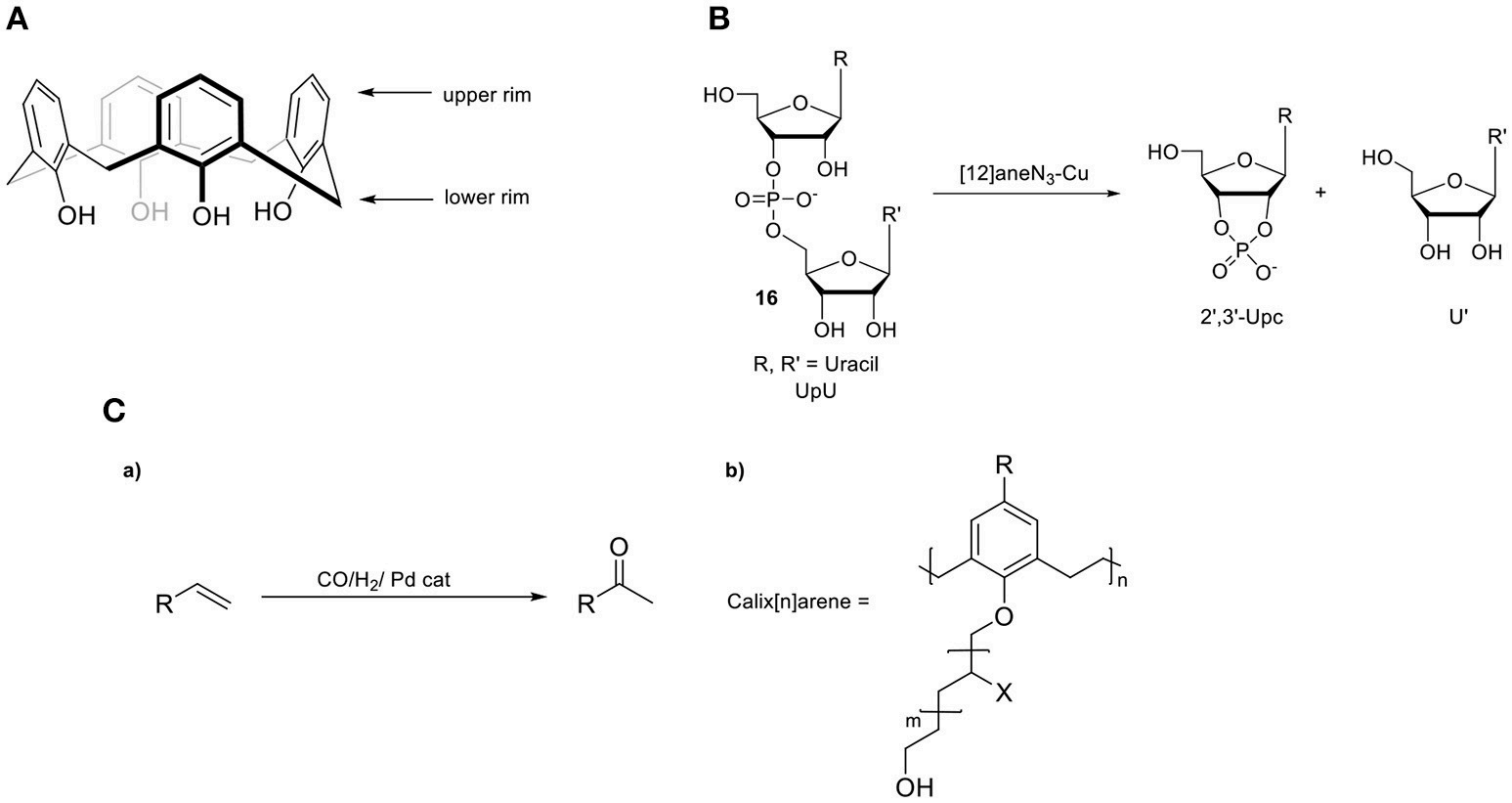
**(A)** Calixarene backbone. **(B)** Intramolecular transesterification of UpU (**16**) by a copper calixarene catalyst. **(C,a)** Wacker oxidation and **(b)** the applied calix[n]arene.

**Figure 10:**
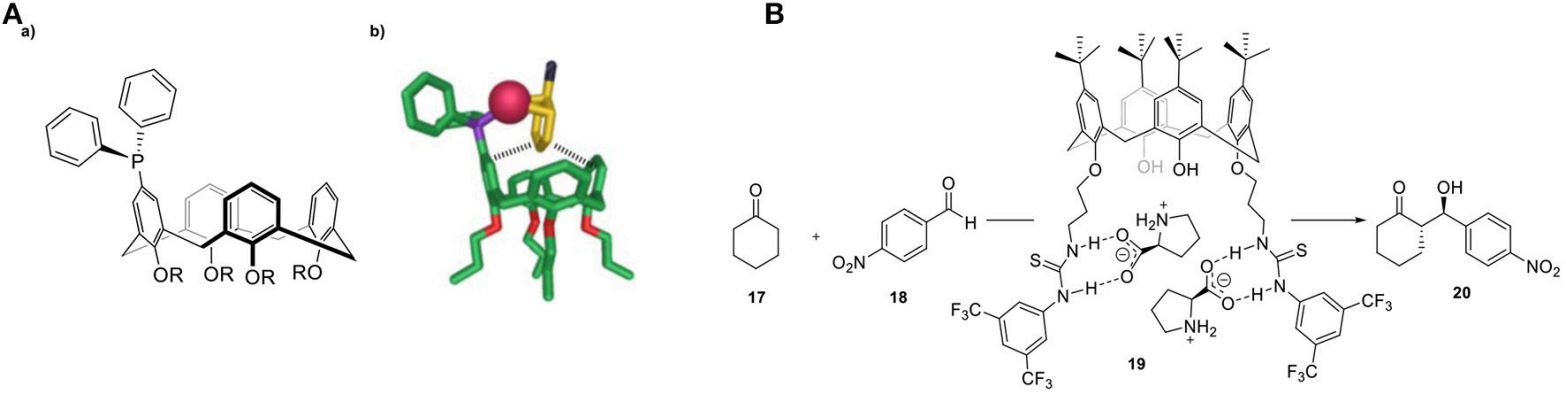
**(A,a)** Applied calix[4]arene ligand in coupling reactions and **(b)** proposed [M(0)(ArX)L] intermediate formed prior to the oxidative addition step. **(B)** Asymmetric aldol reaction catalyzed by an *L*-proline-calixarene-derived achiral thiourea host-guest complex (**19**).

Calix[n]arenes are a class of well-studied hosts molecules that are easily prepared and modified (Gutsche and Muthukrishnan, 1978). These cyclic oligomeric systems of 2-methylene-1-phenol consist of a lower hydrophilic rim (the -OH groups) and a hydrophobic upper rim (the aromatics) (Figure 9A). As a consequence, a hydrophobic, electron-rich cavity is created that can be tuned by incorporating bridging groups other than methylene, such as ether (-CH_2_OCH_2_-), thio (-S), and aza [-CH_2_N(R)CH_2_-] linkages, enabling altered architectures and additional binding sites. An illustrative study on the importance of the cavity during catalysis was reported by the group of Reinhoudt (Cacciapaglia et al., 2006). They described the synthesis of calix[4]arenes functionalized with [12]aneN_3_ ligating units, ranging from one to three units and investigated the catalytic performance of the intramolecular transesterification of the diribonucleoside monophosphate UpU (**16**, Figure 9B). The Cu_2_ and Cu_3_ complexes show a 160- and a 200-fold enhanced rate, respectively, compared to the mononuclear Cu_1_-complex, suggestive of a beneficial role of pre-organizing multiple copper metal centers. The architecture of calixarenes can be utilized for selective transformations depending on the size of the particular substrate, as illustrated by the group of Karakhanov, who used a range of water-soluble calix[n]arenes in the Wacker oxidation of linear alkenes by incorporating Pd^2+^ metal ions as the catalytic active sites (Figure 9C) (Maksimov et al., 2004). Calix[6]arene preferred the oxidation of longer linear alkenes with respect to that of the calix[4]arene, indicating that the size of the cavity is connected to substrate selectivity. The influence of the calix[n]arene cavity on activity and selectivity has been studied in several cross-coupling reactions such as Suzuki-Miyaura, Kumada-Tamao-Corriu, and Mizoroki-Heck. A series of calixarenyl-phosphines have been employed in the palladium-catalyzed Suzuki-Miyaura cross-coupling of phenylboronic acid with aryl halides by the group of Matt (Monnereau et al., 2012). These catalysts proved to exhibit excellent and considerably higher activities compared to those of free triarylphosphines. The same group studied the same cavity-based catalysts in nickel-catalyzed Kumada-Tamao-Corriu and Suzuki–Miyaura cross-couplings (Monnereau et al., 2011, 2017). The authors suggested that the respective aryl-halide species in the transient [M(π-ArX)(calix-phosphine)] intermediate undergoes non-covalent interactions in the cavity (Figure 10A). This results in a bulky environment around the active center, which in turn favors the formation of mono-ligand Pd(0) or Ni(0) intermediates, promoting the subsequent oxidative addition step (Monnereau et al., 2013). Recently Demircan and coworkers reported an asymmetric aldol reaction catalyzed by an *L*-proline-calixarene-derived achiral thiourea host-guest complex (**19**, Figure 10B) (Demircan et al., 2014). The thiourea-containing cavity of the calix[4]arene accelerates the reaction, as it stabilizes the transition state by hydrogen bonding. In addition, the formed hydrophobic cavity proves the potential of catalysis in the presence of water, an otherwise difficult process. In the case of non-polar solvents, the catalyst displayed high yields and excellent enantioselectivities (up to 99%) and diastereoselectivities (97:3). In the case where water was present, the diastereoselectivities (65:35) were moderate whereas the enantioselectivities were high (up to 92%). In addition, several other studies in the field of asymmetric catalysis by enantiopure calix[n]arenes have been recently reported (Li et al., 2011, 2017; Karpus et al., 2018; Sahin et al., 2018).

### Cucurbit[n]urils (CBs)

**Figure 11:**
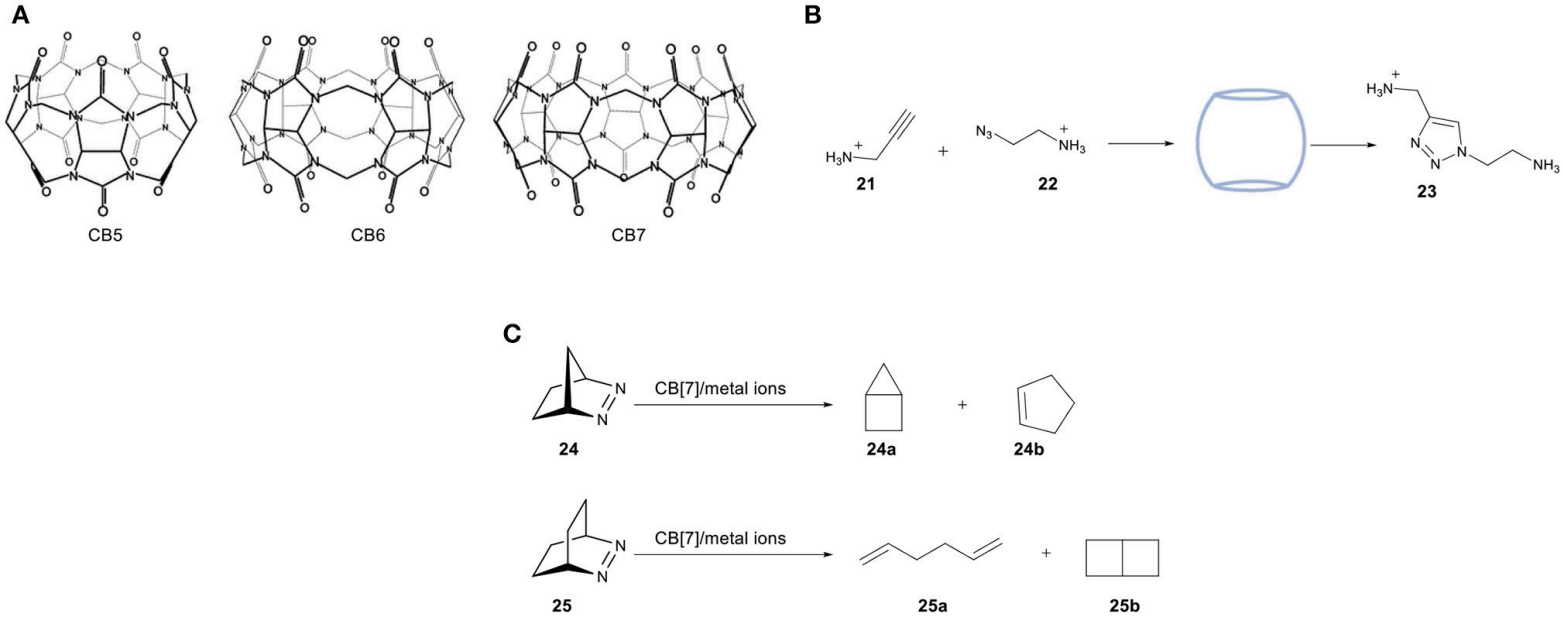
**(A)** Molecular structure of three different CB homologs. **(B)** 1,3-dipolar cycloaddition between azide **21** and alkyne **22** catalyzed by cucurbit[7]uril. **(C)** Chemoselective photoreactions of bicyclic azoalkanes **24** and **25**.

Cucurbiturils (CBs)—the name being derived from their pumpkin-shape structure—are a class of molecular containers that are formed by copolymerization of formaldehyde, glyoxal, and urea (Behrend et al., 1905). After the initial discovery of CB[6] ([6] referring to the number of urea units required for the construction of the overall shape), the chemistry of these water-soluble structures was expanded by preparing different homologs (*n* = 5–10). The formed cavity present in these CBs is similar to that of CDs, as they are made of glycouril units instead of D-glucopyranosyl ones. The cavity size can therefore be adjusted accordingly by increasing the number of glycouril units, a feature that makes them suitable for encapsulating molecules of different molecular sizes (Figure 11A). An interesting property of these macrocycles is their ability to bind both polar and non-polar organic molecules, which stems from the hydrophobic environment inside their cavity combined with the carbonyl groups located at the entrance of the cavity.

A study reported by Mock and coworkers reported the use of CB[6] in the cycloaddition of alkynes (**21**) and azides (**22)**, (Figure 11B) (Mock et al., 1983, 1989). The two substrates have been directly encapsulated inside the host cavity due to the presence of ammonium groups. The 1,3-dipolar cycloaddition is accelerated by a factor of 5.5 × 10^4^ under the catalytic influence of cucurbituril. It has recently been supported by computational studies performed by the group of Maseras (Carlqvist and Maseras, 2007). The high activity was attributed to lower entropic constraints and diminished strain activation of the bound substrates in the cavity. To date, examples of CBs containing transition metal catalysts are limited. One of the most important studies in this field has been reported by Nau and coworkers in 2011 using confined metal ions such as Ti^1+^, Fe^3+^, Co^2+^, Ni^2+^, Cu^2+^, and Ag^1+^ in the chemoselective photoreactions of azoalkanes **24** and **25** (Koner et al., 2011). These reactions can tolerate water, shown by performing the photoreactions in a two-phase mixture of water and pentane. Noteworthy is that the irradiation of **24** under free movement conditions leads to the selective formation of **24a**, whereas irradiation of **25** under the same conditions resulted in a 70:30 mixture of 1,5-hexadiene (**25a**) and bicyclo[2.2.0]hexane (**25b**). Photolysis in the presence of the CB[7]/metal ion system results in an altered selectivity. Photolysis of **24** afforded cyclopentene **24b** as a new photoproduct, whereas photolysis of the bigger azoalkanes **25** afforded a 5-fold to 10-fold excess of the diene product **25a** compared to **25b** (Figure 11C). A study by the group of Herrmann reported a novel design of a three-component supramolecular CB[n]-based system (amino acids, Cu^2+^-ions and CB[8]) that creates a chiral nanoreactor (Zheng et al., 2015). The combination of amino acids as a source of chirality, Cu^2+^ as the catalytically active site and CB[8] as the molecular container resulted in a asymmetric Lewis-acid catalyst for the Diels-Alder reaction of azachalcone with cyclopentadiene. After optimization of the internal volume, this catalytic system is able to catalyze the Diels-Alder reaction with high enantioselectivities (up to 92% ee), whereas the reactions in absence of cavity yields the racemic product. Apart from the high selectivities, improved activities were also obtained. The group of Zhang reported the use of cucurbit[7]uril (CB[7]) in the biphasic oxidation of alcohols by using 2,2,6,6- tetramethylpiperidin-1-oxyl (TEMPO) as the catalyst and NaClO as the oxidant. The proposed first step is the oxidation of TEMPO and the formation of key cationic intermediate (TEMPO^+^), which can undergo unwanted side reactions in water. The use of a supramolecular TEMPO/CB[7] complex resulted in higher conversion to the corresponding aldehyde as a consequence of the electrostatic stabilization of TEMPO^+^ (Jiao et al., 2018).

### Self-Assembled Container Molecules

**Figure 12:**
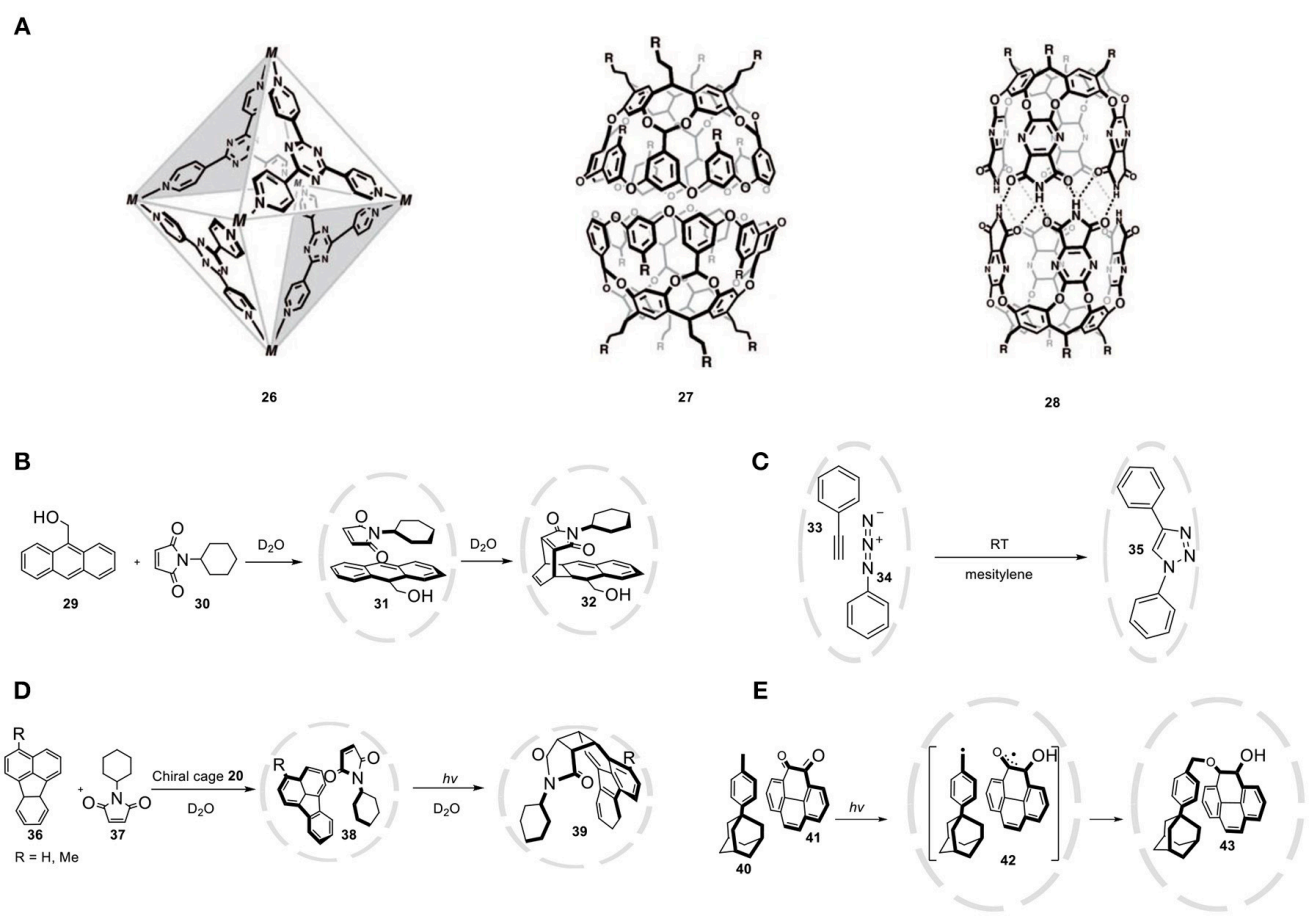
(**A)** Capsules based on non-covalent interactions. **(B)** Diels-Alder reaction between **29** and **30** in the cavity of host **26**, yielding unusual regioisomer **32**. **(C)** A regioselective 1,3-dipolar cycloaddition accelerated within nanoreactor **28**. **(D)** Asymmetric [2+2] photodimerization of fluoranthenes (**36**) and N-cyclohexylmaleimide (**37**) within a chiral analog of cage **26**. **(E)** Regio- and stereoselective bimolecular radical coupling within cage **26**.

Self-assembled capsules are concave building blocks that utilize non-covalent interactions to form a three-dimensional structure with a well-defined nanospace. These bonds can be metal- ligand (**26**) (Fujita et al., 1990), ionic (**27**) (Gibb and Gibb, 2004), or hydrogen-bonding (**28**) (Heinz et al., 1998) interactions that create molecular architectures containing distinct microenvironments (Figure 12A). These interactions can result in discrete, thermodynamically stable capsules with different architectures. An advantage of non-covalent capsules is the relative ease of preparation, ease of variation, and the dynamic properties providing opportunity for guest exchange. The Diels-Alder reaction is a frequently used model reaction applied to evaluate the catalytic properties of the supramolecular hosts. Fujita and coworkers investigated the Diels-Alder reaction of anthracene (**29**) and *N*-cyclohexylmaleimide (**30**) by utilizing a water-soluble organometallic cage (**26**, Figure 12B) as the host (Yoshizawa et al., 2006). Typically anthracene reacts selectively with dienophiles to give the 9,10-adduct. However, due to the steric constraints in the cavity of the host only the terminal anthracene ring reacts, which leads to the formation of the *syn*-adduct (**32**). The finite microenvironment forces the terminal ring of the anthracene to approach the maleimide double bond, thereby pre-organizing the substrate, resulting in 1,4 regioselectivity, which usually is not observed. The selectivity depends on the steric bulk of *N*-propylmaleimide, as less bulky co-reagents lead to lower regioselectivity. Unfortunately, the reaction proved to be non-catalytic due to product inhibition. Rebek reported the use of self-assembled cages as catalyst for a 1,3-dipolar cycloaddition of phenylacetylene (**33)** and phenyl azide (**34**) (Chen and Rebek, 2002). The hydrogen bonding-based cage **28** encapsulates the two guest molecules in a edge-to-edge fashion, which is dictated by the constrained environment of the cavity. The resulting proximity effect accelerates the cycloaddition by 30,000-fold. The confinement not only leads to rate enhancement but also to the exclusive formation of the 1,4-triazole (**35**, Figure 12C). Performing this 1,3-dipolar cycloaddition in bulk solution yields a 1:1 mixture of 1,2 and 1,4 cycloadduct. The authors have suggested that the observed rate acceleration is due to the effective concentration within the cavity, whereas the high regioselectivity is a result of substrate pre-organization inside the capsule. Lastly, nanoreactor **28** proved to allow for size-selectivity, given that the use of larger azide substrates did not result in reaction rate acceleration.

The group of Fujita reported the synthesis of a chiral hetero-coupling product by performing the reaction within a chiral environment (Nishioka et al., 2008). The chiral cage was prepared by replacing the ethylenediamine end caps on the metal nodes with enantiopure chiral diamines. Even though the chiral moieties are located away from the space where the reaction takes place, the reaction of fluoranthene **(36)** and *N*-cyclohexylmaleimide **(37)** results in the formation of the desired product **39** with an enantiomeric excess of 40% ee, and even 50% ee when R = Me (Figure 12D). Regio- and stereo-selectivity has been clearly demonstrated in bimolecular radical reactions as well, where controlling the selectivity of this reaction proved to be difficult. By taking advantage of the size and shape-selectivity of cage **26**, photoexcitation of *o*-quinone (**41**) and a very sterically hindered toluene derivative **40** selectively afforded the 1,4 adduct **43** in 70% yield (Figure 12E) (Yamaguchi and Fujita, 2008). The reactivity of the formed benzylic and semiquinone radicals (**42**) is controlled by cage **26**, which induces selective cross-coupling. Performing the same reaction without cage **26**, an unidentified mixture of products was formed that did not contain the 1,4 adduct, demonstrating that cage **26** accelerates the O-coupling pathway and at the same time suppresses other competitive pathways.

**Figure 13:**
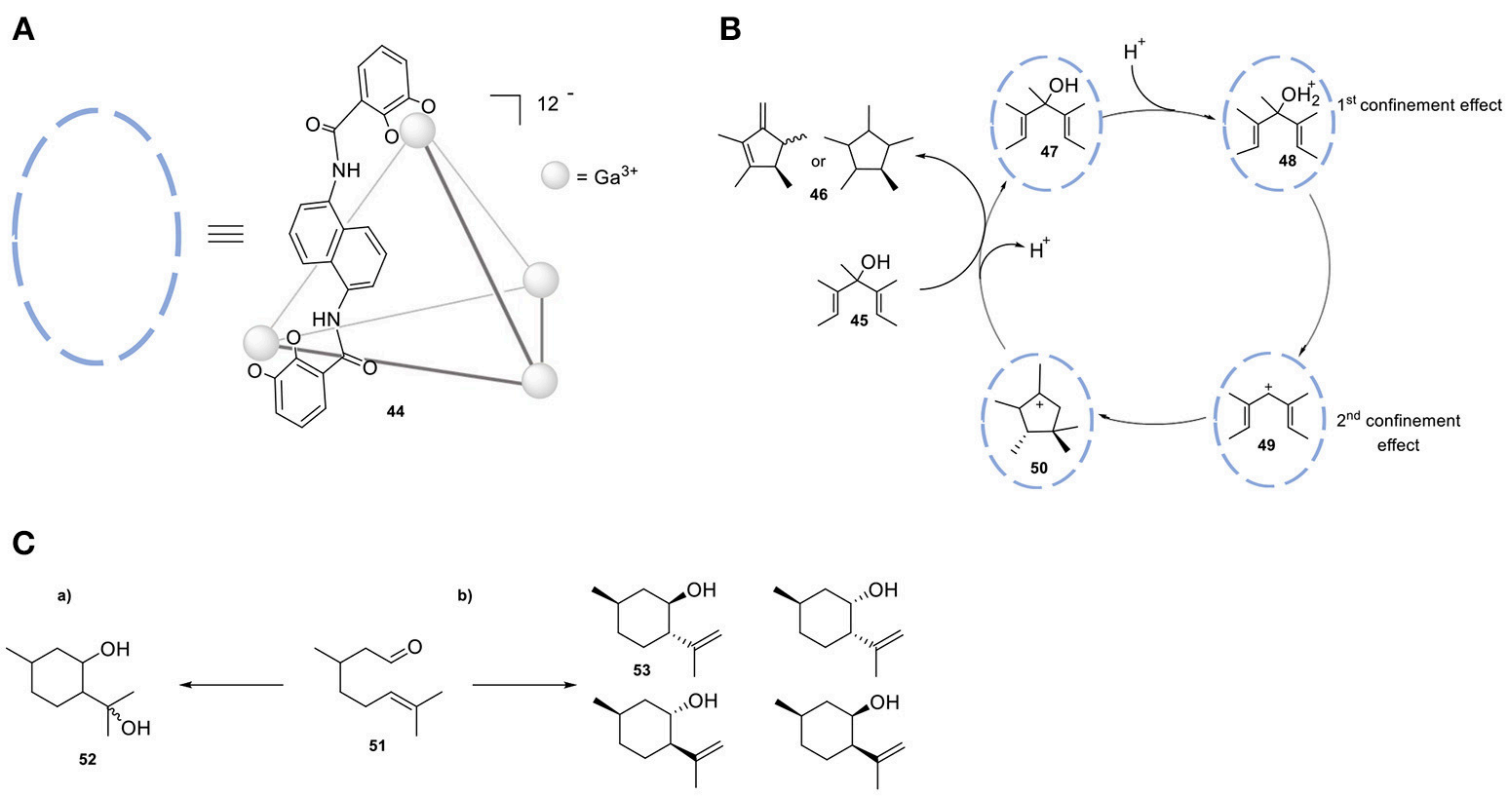
**(A)** Schematic model of tetrahedral cage **35** (Ga_4_L${}_{6}^{12-}$); only one ligand L is displayed as chemdraw structure for clarity. **(B)** Proposed catalytic mechanism of Nazarov of 1,3-pentadienols by utilizing cage **35**. **(C)** Cyclization of monoterpene citronellal in the absence **(a)** and in the presence **(b)** of cage **35**.

Cage **44**, [Ga_4_L_6_]${}_{,}^{12-}$ shows a strong preference for cationic guests (Figure 13A). The high anionic charge leads to a high local pH, which can be employed in the protonation of weak basic guest species and ultimately leads to acid-mediated catalysis. The group of Raymond investigated the proton-catalyzed hydrolysis of orthoformates. While the reactivity of orthoformates in a neutral or basic aqueous solution is relatively low, encapsulation in cage **44** leads to the protonation of an ethereal oxygen by stabilizing the conjugate acid of these guest molecules. Upon confinement-assisted protonation, hydrolysis leads to the corresponding carboxylic ester, followed by acid- or base-catalyzed hydrolysis of the ester. The desired product carries an anionic charge, leading to facile expulsion from the cage cavity. This property of cage **44** has been combined with size-selectivity; only orthoformates smaller than tripentyl orthoformate were readily hydrolyzed with 1 mol % of **44**. Raymond et al. also applied the same strategy in the Nazarov cyclization of 1,3-pentadienols (**45**, Figure 13B) to form cyclopentadienes (**46**) (Hastings et al., 2010). In this study cage **44** has a bifunctional role, because it not only facilitates the protonation of a weak basic alcohol (**47**) but also favors the subsequent electrocyclization of the dienyl cation intermediate **48**. The protonation is favored in the cavity of the host and the subsequent cyclization is driven by substrate pre-organization. The transition state for the cation cyclization is stabilized by cation-π interactions in the cavity of the supramolecular host **44**. The rate accelerations of the catalyzed reaction in relation to the uncatalyzed reaction are in the order of 10^6^. The same group reported the catalytic cyclization of the monoterpene citronellal **(51)** by performing the reaction in the presence of the same cage **(44)** (Hart-Cooper et al., 2012). It has been found that the presence of **44** leads to the formation of **53** as the major product, while the non-confined Brønsted acid catalyst forms the diol **52** (Figure 13C). Several analogs of cage **44** have been employed in order to evaluate the relation between the host architecture and selectivity in catalysis (Hart-Cooper et al., 2015). Varying the ditopic ligand of the host did not alter the product selectivity, whereas modification of the size of the cavity resulted in significant changes in activity and product selectivity. Turnover numbers of up to 840 and rate accelerations in the order of 105-fold relative to the uncatalyzed reaction have been measured (Slagt et al., 2001, 2004; Jacobs et al., 2013).

**Figure 14:**
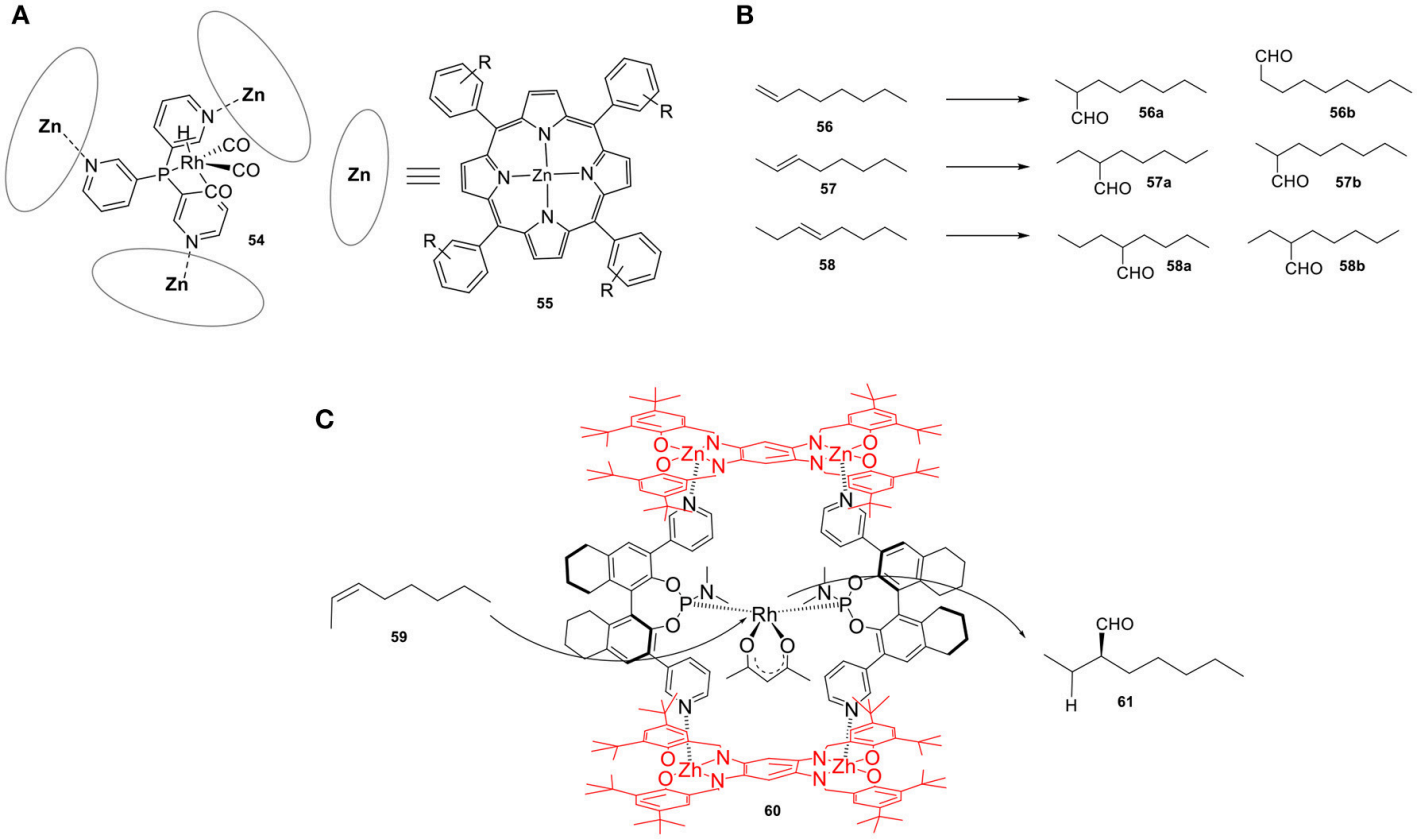
**(A)** Confined Rh catalyst by three zinc(II)-porphyrin building units (**42**). **(B)** Products obtained in alkene hydroformylation. **(C)** A supramolecular chiral rhodium catalyst employed in the asymmetric hydroformylation of internal alkenes.

Reek and coworkers developed a versatile Rh catalyst (**54**) for the hydroformylation of alkenes (Figure 14A). Confinement of an active Rh catalyst using a tris(pyridyl)phosphine and three zinc(II)-porphyrin building units, which gives rise to a capsule-shaped ligand sphere, resulted in a highly active catalyst that led to unusual branched selectivity in the hydroformylation of 1-octene (**56**). The application of the same catalytic system in the hydroformylation of internal alkenes gave rise to products **57a** and **58a** (Figure 14B) (Kuil et al., 2006). These results were rationalized by both theoretical and experimental investigations. The step that determines the selectivity turned out to be the hydride migration step and the fact that this step takes place within the cavity of the host results in different selectivity. The restricted environment suppresses competing reaction pathways that lead to the other regio-isomer explaining the formation of C3-aldehyde in high selectivity. An additional feature of this catalytic system is the strong correlation between the selectivity and the structure of the cavity, as minor modifications to the porphyrins result in dramatic changes in the observed selectivity. This feature has been demonstrated by the replacement of zinc-porphyrins with zinc-phthalocyanines as building blocks (Bocokić et al., 2013), which leads to a cavity with a larger volume that in turn alters the selectivity toward the formation of **57b** and **58b**. In this perspective, as the catalyst is the same, the second coordination sphere dictates the regioselectivity of this reaction, providing access to internal aldehydes in selectivities that are difficult to achieve by traditional strategies. An encapsulated Rh catalyst for the asymmetric hydroformylation of internal alkenes was also developed by the same group (Bellini and Reek, 2012) by incorporating bulky zinc-based building blocks onto chiral bipyridine phosphoramidite ligands (**60**). Upon coordination, the binding mode of the ligand changes from equatorial to axial, *trans* to the hydride. The resulting catalytic system proved to be efficient in the asymmetric hydroformylation of internal alkenes, e.g., 2-octene. When using the non-confined *cis*-catalyst, the same reactions resulted in a low conversion (12%) of 2-octene (**59**) and low ee (25%) of the C3-aldehyde (**61**). On the other hand, utilizing the confined catalyst led to the formation of the *trans*-complex with a substantial increase of both conversion (56%) and ee (45%). Interestingly, by using a more rigid host structure (Figure 14C), a well-defined chiral space was formed around the bis-chelated rhodium catalyst, which finally led to higher regioselectivity and enantioselectivity (Gadzikwa et al., 2012). An enantiomeric ratio of up to 93:7 was measure for the C3-aldehyde. The same laboratory reported a regio- and enantioselective catalyst for the asymmetric hydroformylation of styrene that showed much larger enantioselectivities (García-Simón et al., 2015). The entrapment of a Rh catalyst in a supramolecular metallocage converts styrene derivatives into the corresponding aldehyde products with enantiomeric excess of up to 74%. The degree of chiral induction observed is much higher than that of the non-encapsulated Rh catalyst. Based on calculations, the absence of confinement has a detrimental effect on the enantioselectivity because styrene can coordinate in four different orientations to the two available coordination sites, leading to poor stereoselectivity. On the other hand, when the catalysis takes place in the confined space, the restricted environment hinders most of the coordination modes to the catalytic center and therefore suppresses competitive reaction pathways. The authors showed via spectroscopic investigations that the electronic effects of the catalyst and its first coordination sphere remain the same upon encapsulation.

**Figure 15:**
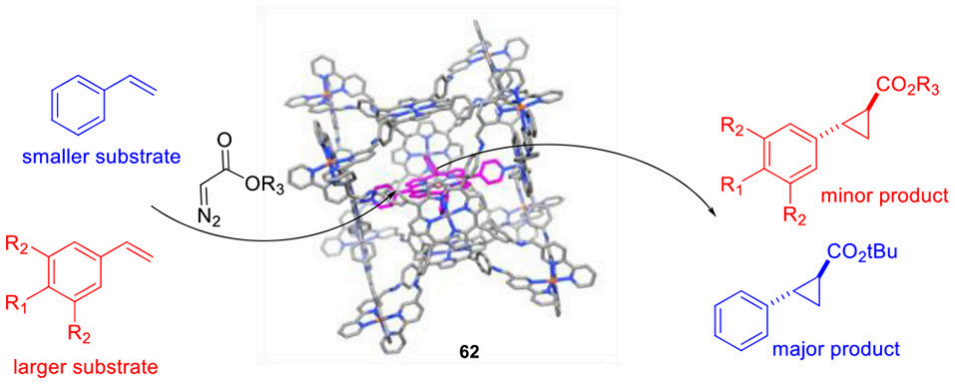
Size-selectivity in cyclopropanation reactions by with an encapsulated cobalt-porphyrin catalyst (**62**).

De Bruin and coworkers studied the supramolecular encapsulation of a cobalt-porphyrin catalyst for the cyclopropanation of styrene (Otte et al., 2013, 2014). The major deactivation pathways of such radical-type transition metal catalysis involve the formation of bimetallic species and the prevention of such pathways will be fruitful in terms of catalytic activity and selectivity. One way to suppress this unwanted side reaction is the encapsulation of a single catalytically active cobalt(II)-*tetra*pyridylporphyrin inside a cubic M_8_L_6_ cage (**62**), leading to “site-isolation” of the Co-species. The encapsulated cobalt catalysts showed excellent activities that can even compete with the activity of the best cobalt(II)-porphyrin currently used for this kind of catalytic transformations in polar solvents. In addition, the catalyst proved to be a highly versatile system thanks to its compatibility with a wide range of styrene analogs bearing either electron-donating or electron-withdrawing groups. Subsequent research revealed that the encapsulated cobalt(II)-porphyrin catalyst is size-selective for smaller styrene analogs (Figure 15). Additionally, encapsulation increases the water-solubility of the transition metal complex.

**Figure 16:**
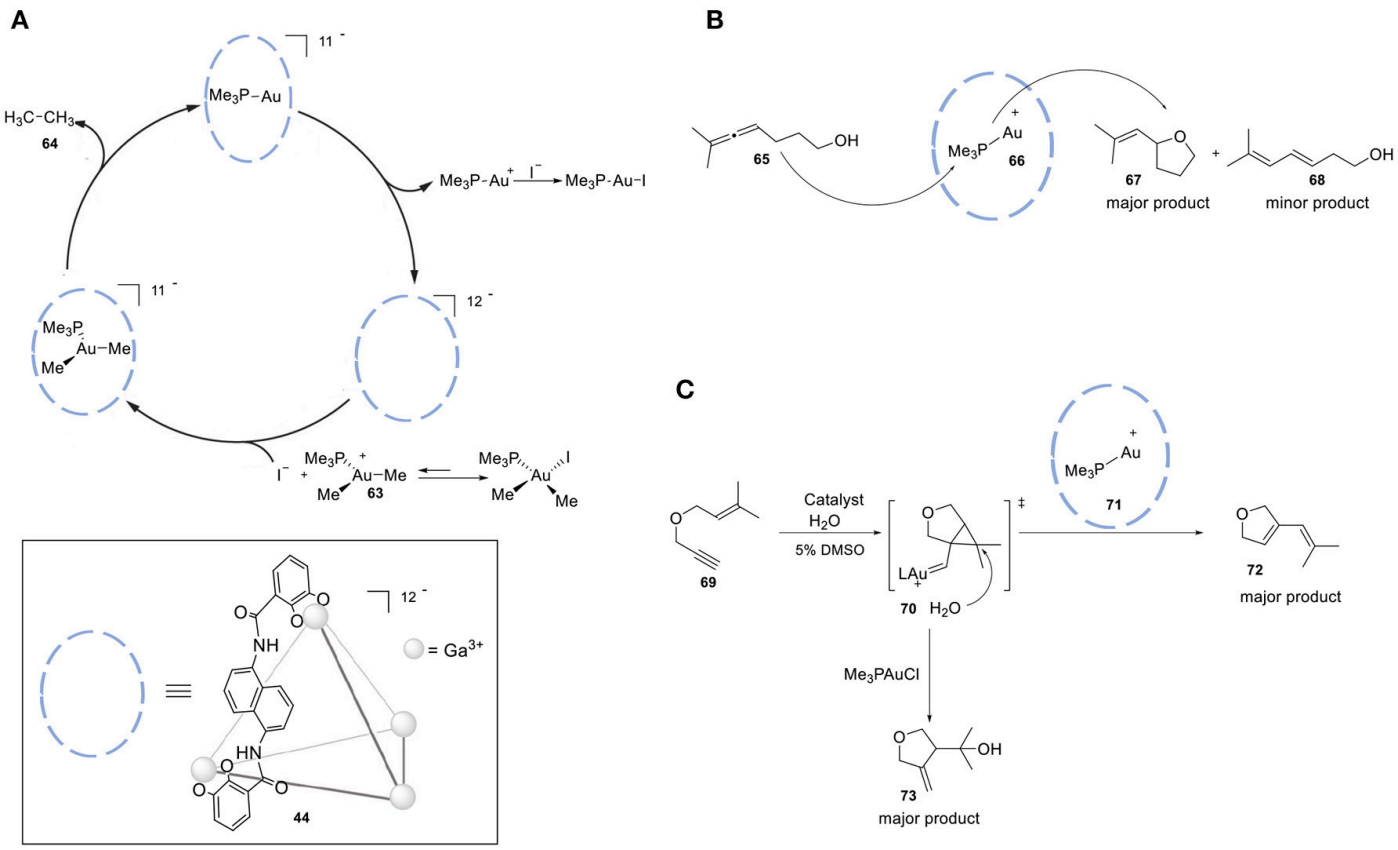
**(A)** Catalytic alkyl-alkyl cross-coupling by utilizing a highly anionic supramolecular catalyst. **(B)** Selective hydroalkoxylation of allene **52**. **(C)** Catalysis of the intramolecular cyclo-isomerization reaction by the encapsulated gold complex compared to the free gold catalyst.

**Figure 17:**
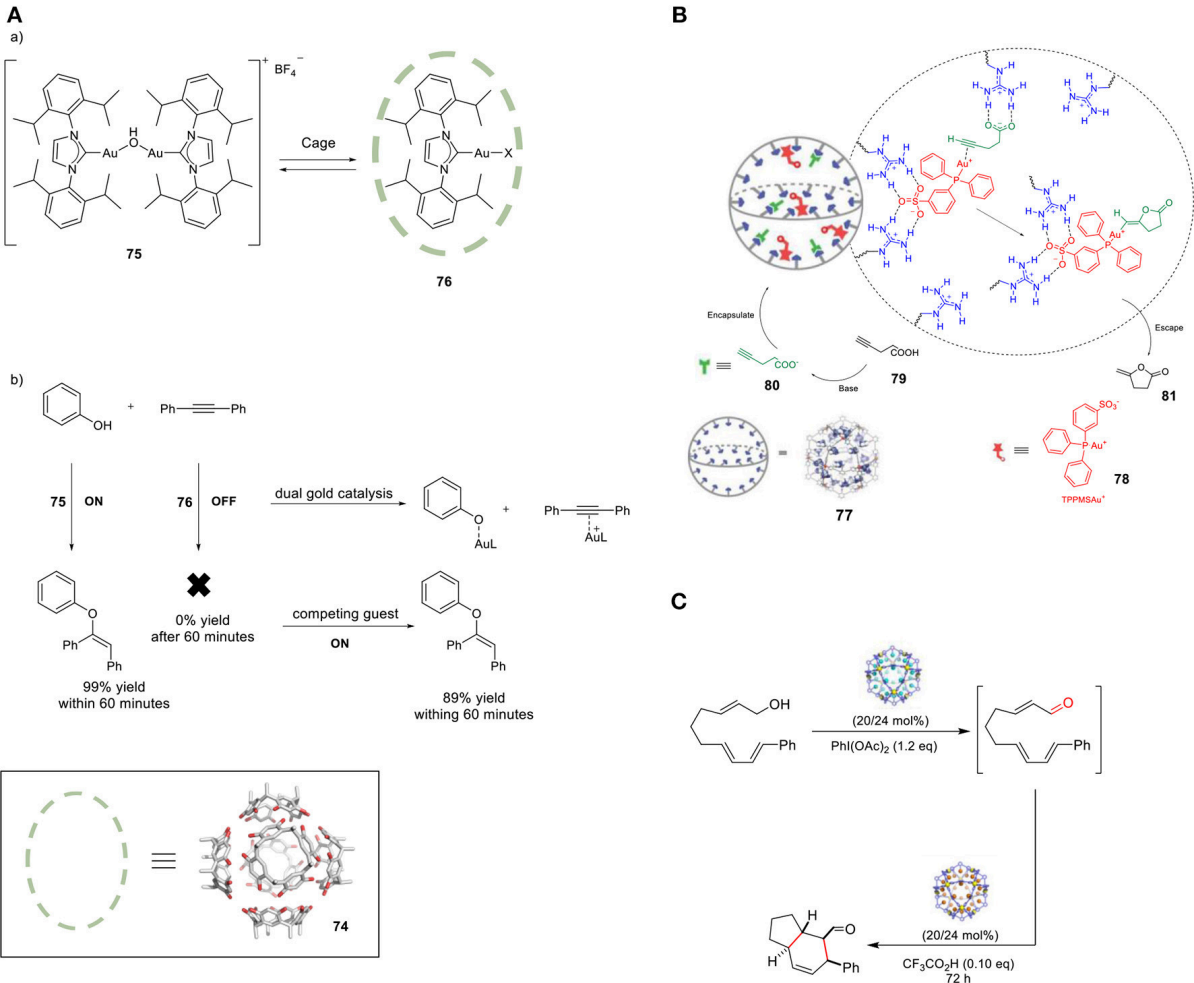
**(A,a)** Encapsulation of [{Au(IPr)}_2_(μ-OH)](BF_4_) in hexameric resorcin[4]arene cage. **(b)** Switching the gold-catalyzed hydrophenoxylation off and on. **(B)** Schematic representation of the base-triggered catalytic gating process. **(C)** Self-Assembled molecular containers for catalyst isolation enabling two-step cascade reactions. Reproduced with permission from Ueda et al. (2017).

The groups of Raymond and Bergman reported increased activity in the Au-mediated alkyl-alkyl cross-coupling reaction upon encapsulation (Kaphan et al., 2015). The combination of supramolecular assembly (**44**) with a transition metal catalyst successfully promoted this challenging C-C bond forming reaction. In particular, the applied host is anionic and, in combination with its hydrophobic cavity, this feature favors the encapsulation of cationic species. The authors brought forth a different mechanism than generally proposed for this class of reactions. Interestingly, halide dissociation takes place prior to encapsulation, which generates a transient cationic gold(III) dialkyl complex (**63**) trapped inside the anionic cage. The restricted microenvironment of the cage increases the rate of the reductive elimination, leading to formation of the desired cross-coupled product **64** (Figure 16A). In addition, size-selectivity for the substrate was observed. The catalytic performance of this system was evaluated in intramolecular cyclization reactions. More specifically, the encapsulated gold catalyst was used in the hydroalkoxyation of allene (**65**) for the selective formation of allylic ether (**67**, Figure 16B). Confinement led to an enhancement in the catalytic activity of the transition metal complex and improvement of the observed chemo- and regioselectivity. Lastly, the hydrophobic cavity of cage **44** enabled the use of water, avoiding the typically required organic solvents for this reaction. The application of the encapsulated gold catalyst **71** in the cyclo- isomerization of enyne **(69)** resulted in the formation of a different product with respect to the one formed by the free complex (Hart-Cooper et al., 2012), proposely because of the hydrophobicity of the cavity. When the reaction occurs in the bulk, water attacks the well-solvated carbene species **70**, which leads to the formation of the hydroalkoxylated species **73**. When the reaction occurs inside the cage, the hydrophobic cavity partially protects the carbenium ion intermediate, giving it enough time to form product **72** via cyclo-isomerization (Figure 16C). Recently, the same group investigated the stabilizing role of the charge in supramolecular catalysis by developing an isostructural octaanionic version of the cage **44** (Hong et al., 2018). They experimentally showed the stabilization effect of the anionic charge of the cage in the Nazarov cyclization. The rate constant was 680 times higher in the case of the dodecaanionic catalyst, highlighting the importance of charge and electrostatic effect in catalysis. Reek and coworkers reported a switchable gold catalyst by encapsulation in a self-assembled hexameric resorcin[4]arene cage **74** (Figure 17A) (Jans et al., 2016). In the presence of the cage, the dinuclear complex [{Au(NHC)}_2_(μ-OH)](X) (**75**) is broken up and encapsulated as mononuclear species, resulting in site isolation of the mononuclear gold complex (**76**), which prohibited the typical σ,π- dual activation mode and led to typical behavior because of mononuclear activation. The chemoselectivity could be switched to the digold- mediated reaction by adding a competing guest that would bind more strongly in the cage than the gold catalyst. The same group reported an interesting study demonstrating the positive effect of confining a catalyst in terms of reaction rates (Figure 17B) (Wang et al., 2016). M_12_L_24_ sphere **77** (M = Pd, L = organic bipyridine ligand) functionalized with 24-fold endohedral guanidinium-binding motifs displays strong binding of sulfonate guest by cooperative hydrogen bonding; carboxylate containing guests are also encapsulated, albeit less strongly. TPPMSAu^+^ catalysts **78** were strongly bound within the sphere and acetylenic carboxylate substrates (**80**) were pre-organized by adjacent guanidinium sites for an efficient cyclization to give the enol lactone (**81**). In a follow-up study the same sphere was utilized for the encapsulation of a sulfonate-functionalized ruthenium water oxidation catalyst (Yu et al., 2018). Preorganization of dilute solutions of sulfonate-functionalized ruthenium complexes led to high high local catalyst concentrations. The preorganization effect enhances the water oxidation rate by two-orders of magnitude. An example of a site-isolated cascade reaction is from Fujita and coworkers (Ueda et al., 2017). Herein the authors could synthesize and mix two different M_12_L_24_ supramolecular spheres in one pot, each sphere being decorated on the inside with their own specific catalyst. This system performs an oxidation followed by Diels-Alder reaction in tandem, whereas mixing the naked catalysts (without their metal-ligand shell) results in no formation of the final product (Figure 17C).

## Conclusions and Perspectives

Chemists have been inspired by enzymes ever since their working principles became clear. One of the essential elements is the specific cage around the active site, which in enzymes is defined by the peptide environment. Generating well-defined cages around catalytic active sites has been challenging for years, but there is a lot of progress in recent years. Interestingly, the generation of well-defined cages around catalytic active centers has been pursued for both homogeneous and heterogeneous catalysts. For the latter, many structured solid materials have been developed, with zeolites playing an important role in catalysis. The initial focus in the area of homogeneous catalysis was predominantly on the study of covalent hosts such as cyclodextrins, calixarenes, cucurbiturils, and other macrocyclic molecular architectures. More recently, a lot of self-assembled cages and coordination cages have been used, tremendously increasing opportunity space. Moreover, metal-organic frameworks have attracted much attention, at least partly related to the high degree of tunability provided by this class of well-defined structured materials. This nicely links the fields of homogeneous and heterogeneous approaches, as both coordination cages and MOFs are generated from organic building blocks and metal precursors. There are now many examples that demonstrate that a well-defined cage-like porous structure has a great influence on the activity and selectivity of the catalyst. These confinement effects can be based on entropy effects, cage-wall effects, absorption, desorption, shape-, and size-selectivity, as typically observed in the field of zeolites. The cage environment also results in geometrical constraints, which may lead to typical folding of substrate explaining some of the selectivity effects. Changes in the potential energy surface have also been reported, and this may be more often the focus of cages that are used to facilitate organic transformations.

Next to these positive effects on the catalytic events, caged catalysts may also experience limitations. For example, the size of the entrance to the active sites present in molecular cages, MOFs or zeolites may limit the substrate-scope by size or may reduce reaction rates due to diffusion limitations. Product inhibition can also be a major issue when the cage has a larger affinity for the product than the substrate. This has been observed for some of the organic transformations in molecular cages. Therefore, the ability to design molecular hosts with a wide range of apertures and cage sizes is of utmost importance.

For the future, better understanding of confinement effects in catalysis is required to further facilitate tailor-made design strategies. With this review we show that there are opportunities for the fields of homogeneous and heterogeneous catalysis to work in a constructive manner to gain further insights. For practical applications it is important to develop systems that can be prepared at sufficient scale at reasonable costs. Overall, we are optimistic and believe that catalysis in confined spaces will become even more important as a research field.

## Author Contributions

All authors listed have made a substantial, direct and intellectual contribution to the work, and approved it for publication.

### Conflict of Interest Statement

The authors declare that the research was conducted in the absence of any commercial or financial relationships that could be construed as a potential conflict of interest.

## References

[B1] Ahmad N. Chughtai A. H. Younus H. A. Verpoort F. (2014). Discrete metal-carboxylate self-assembled cages: design, synthesis and applications. Coord. Chem. Rev. 280, 1–27. 10.1016/j.ccr.2014.07.005

[B2] Alber T. Gilbert W. A. Ponzi D. R. Petsko G. A. (1983). The role of mobility in the substrate binding and catalytic machinery of enzymes. Ciba Found. Symp. 93, 4–24. 10.1002/9780470720752.ch2 6551232

[B3] Ayad Z. S. (2002). Aromatization of n-hexane over zeolite catalysts of varying silica alumina ratio. JEAS. 49, 387–403.

[B4] Banerjee R. Phan A. Wang B. Knobler C. Furukawa H. O'Keeffe M.. (2008). High-throughput synthesis of zeolitic imidazolate frameworks and application to CO2 capture. Science 319, 939–943. 10.1126/science.1152516 18276887

[B5] Batten S. R. Hoskins B. F. Robson R. (1995). Two Interpenetrating 3D networks which generate spacious sealed-off compartments enclosing of the order of 20 solvent molecules in the structures of Zn(CN)(NO3)(tpt)2/3.cntdot.solv (tpt = 2,4,6-tri(4-pyridyl)-1,3,5-triazine, solv =.apprx.3/4C2H2Cl4.cntdo. J. Am. Chem. Soc. 117, 5385–5386. 10.1021/ja00124a032

[B6] Behrend R. Meyer E. Rusche F. I. (1905). Condensationsproducte aus Glycoluril und Formaldehyd. Justus Liebig's Ann Chem. 339, 1–37. 10.1002/jlac.19053390102

[B7] Bell A. T. (2003). The impact of nanoscience on heterogeneous catalysis. Science 299, 1688–1691. 10.1126/science.1083671 12637733

[B8] Beller M. (2011). Preface for the themed issue of Chemical Society Reviews. Chem. Soc. Rev. 40, 4891–4892. 10.1039/C1CS90038B 21892452

[B9] Bellini R. Reek J. N. (2012). Coordination studies on supramolecular chiral ligands and application in asymmetric hydroformylation. Chemistry 18, 7091–7099. 10.1002/chem.201200225 22532382

[B10] Bocokić V. Kalkan A. Lutz M. Spek A. L. Gryko D. T. Reek J. N. H. (2013). Capsule-controlled selectivity of a rhodium hydroformylation catalyst. Nat. Commun. 42670, 1–9. 10.1038/ncomms 24150228

[B11] Breslow R. (1995). Biomimetic chemistry and artificial enzymes: catalysis by design. Acc. Chem. Res. 33, 146–153. 10.1021/ar00051a008

[B12] Breslow R. Campbell P. (1969). Selective aromatic substitution within a cyclodextrin mixed complex. J. Am. Chem. Soc. 91, 3085–3092. 10.1021/ja01039a044

[B13] Breslow R. Overman L. E. (1970). “Artificial enzyme” combining a metal catalytic group and a hydrophobic binding cavity. J. Am. Chem. Soc. 92, 1075–1077. 10.1021/ja00707a062 5451011

[B14] Côté A. P. Benin A. I. Ockwig N. W. O'Keeffe M. Matzger A. J. Yaghi O. M. (2005). Porous, crystalline, covalent organic frameworks. Science 310, 1166–1170. 10.1126/science.1120411 16293756

[B15] Cacciapaglia R. Casnati A. Mandolini L. Reinhoudt D. N. Salvio R. Sartori A.. (2006). Catalysis of diribonucleoside monophosphate cleavage by water soluble Copper(II) Complexes of Calix[4]arene based nitrogen ligands. J. Am. Chem. Soc. 128, 12322–12330. 10.1021/ja0632106 16967984

[B16] Canivet J. Aguado S. Daniel C. Farrusseng D. (2011). Engineering the Environment of a Catalytic Metal-Organic Framework by Postsynthetic Hydrophobization. Chem. Cat. Chem. 3, 675–678. 10.1002/cctc.201000386

[B17] Carlqvist P. Maseras F. (2007). A theoretical analysis of a classic example of supramolecular catalysis. Chem. Commun. 7, 748–750. 10.1039/b613434c 17392971

[B18] Cejka J. Corma A. Zones S. (2010). Zeolites and Catalysis: Synthesis, Reactions and Applications. KGaA, Weinheim: WILEY-VCH Verlag GmbH & Co.

[B19] Chen J. Rebek J. (2002). Selectivity in an encapsulated cycloaddition reaction. Org. Lett. 4, 327–329. 10.1021/ol0168115 11820871

[B20] Cho H. Y. Yang D. A. Kim J. Jeong S. Y. Ahn W. S. (2012). CO_2_ adsorption and catalytic application of Co-MOF-74 synthesized by microwave heating. Catal. Today 185, 35–40. 10.1016/j.cattod.2011.08.019

[B21] Climent M. Corma A. Velty A. Susarte M. (2000). Zeolites for the Production of fine chemicals: synthesis of the fructone fragrancy. J. Catal. 196, 345–351. 10.1006/jcat.2000.3044

[B22] Corma A. (2003). State of the art and future challenges of zeolites as catalysts. J. Catal. 216, 298–312. 10.1016/S0021-9517(02)00132-X

[B23] Corma A. Díaz-Cabañas M. J. Jordá J. L. Martínez C. Moliner M. (2006). High-throughput synthesis and catalytic properties of a molecular sieve with 18-and 10-member rings. Nature 443, 842–845. 10.1038/nature05238 17051215

[B24] Cornils B. Herrmann W. A. (2002). Applied Homogeneous Catalysis with Organometallic Compounds. KGaA, Weinheim: WILEY-VCH Verlag GmbH & Co.

[B25] Crini G. (2014). Review: A history of cyclodextrins. Chem. Rev. 114 10940–10975. 10.1021/cr500081p 25247843

[B26] Cundy C. S. Cox P. (2005). The hydrothermal synthesis of zeolites: precursors, intermediates and reaction mechanism. Microporous Mesoporous Mater. 82, 1–78. 10.1016/j.micromeso.2005.02.016

[B27] De Clercq R. Dusselier M. Makshina E. Sels B. F. (2018). Catalytic Gas-Phase Production of Lactide from Renewable Alkyl Lactates. Angew. Chem. Int. Ed. 57, 3074–3078. 10.1002/anie.201711446 29356294

[B28] Demircan E. Eymur S. Demir A. S. (2014). Proline–calixarene thiourea host–guest complex catalyzed enantioselective aldol reactions: from nonpolar solvents to the presence of water. Tetrahedron 25, 443–448. 10.1016/j.tetasy.2014.01.015

[B29] Deng H. Grunder S. Cordova K. E. Valente C. Furukawa H. Hmadeh M.. (2012). Large-pore apertures in a series of metal-organic frameworks. Science 336, 1018–1023. 10.1126/science.1220131 22628651

[B30] Derouane E. G. (1997). Zeolites as solid solvents. J. Mol. Catal. A Chem. 134, 29–45. 10.1016/S1381-1169(98)00021-1

[B31] Diercks C. S. Yaghi O. M. (2017). The atom, the molecule, and the covalent organic framework. Science 355, 1–8. 10.1126/science.aal1585 28254887

[B32] Ding S. Y. Gao J. Wang Q. Zhang Y. Song W. G. Su C. Y.. (2011). Construction of covalent organic framework for catalysis: Pd/COF-LZU1 in Suzuki–miyaura coupling reaction. J. Am. Chem. Soc. 133, 19816–19822. 10.1021/ja206846p 22026454

[B33] Dodziuk H. (2006). Cyclodextrins and Their Complexes: Chemistry, Analytical Methods, Applications. KGaA, Weinheim: WILEY-VCH Verlag GmbH & Co.

[B34] Dusselier M. Davis M. E. (2018). Small-pore zeolites: synthesis and catalysis. Chem. Rev. 116, 5265–5329. 10.1021/acs.chemrev.7b00738 29746122

[B35] Dusselier M. Van Wouwe P. Dewaele A. Jacobs P. A. Sels B. F. (2015). Shape-selective zeolite catalysis for bioplastics production. Science 349, 78–80. 10.1126/science.aaa7169 26138977

[B36] El-Kaderi H. M. Hunt J. R. Mendoza-Cortés J. L. Côt,é A. P. Taylor R. E. O'Keeffe M.. (2007). Designed synthesis of 3D covalent organic frameworks. Science 316, 268–272. 10.1126/science.1139915 17431178

[B37] Falkowski J. M. Sawano T. Zhang T. Tsun G. Chen Y. Lockard J. V.. (2014). Privileged phosphine-based metal-organic frameworks for broad-scope asymmetric catalysis. J. Am. Chem. Soc. 136, 5213–5216. 10.1021/ja500090y 24684238

[B38] Fang Q. Gu S. Zheng J. Zhuang Z. Qiu S. Yan Y. (2014). 3D microporous base-functionalized covalent organic frameworks for size-selective catalysis. Angew. Chem. Int. Ed. 53, 2878–2882. 10.1002/anie.201310500 24604810

[B39] Farha O. K. Eryazici I. Jeong N. C. Hauser B. G. Wilmer C. E. Sarjeant A. A.. (2012). Metal-organic framework materials with ultrahigh surface areas: Is the sky the limit? J. Am. Chem. Soc. 134, 15016–15021. 10.1021/ja3055639 22906112

[B40] Farkas G. Császár Z. Balogh S. Szöllosy Á. Gouygou M. Bakos J. (2013). Phosphine-phosphite ligands in the palladium-catalyzed asymmetric allylic alkylation: electronic and steric effects. Catal. Commun. 36, 94–97. 10.1016/j.catcom.2013.03.005

[B41] Feng D. Gu Z. Y. Li J. R. Jiang H. L. Wei Z. Zhou H. C. (2012). Zirconium-metalloporphyrin PCN-222: mesoporous metal-organic frameworks with ultrahigh stability as biomimetic catalysts. Angew. Chem. Int. Ed. 51, 10307–10310. 10.1002/anie.201204475 22907870

[B42] Ferreira K. N. Iverson T. M. Maghlaoui K. Barber J. Iwata S. (2004). Architecture of the photosynthetic oxygen-evolving center. Science 303, 1831–1838. 10.1126/science.1093087 14764885

[B43] Fujita M. Yazaki J. Ogura K. (1990). Preparation of a macrocyclic polynuclear complex, [(en)Pd(4,4'-bpy)]4(NO3)8 (en = ethylenediamine, bpy = bipyridine), which recognizes an organic molecule in aqueous media. J. Am. Chem. Soc. 112, 5645–5647. 10.1021/ja00170a042

[B44] Furukawa H. Go Y. B. Ko N. Park Y. K. Uribe-Romo F. J. Kim J.. (2011). Isoreticular expansion of metal-organic frameworks with triangular and square building units and the lowest calculated density for porous crystals. Inorg. Chem. 50, 9147–9152. 10.1021/ic201376t 21842896

[B45] Gadzikwa T. Bellini R. Dekker H. L. Reek J. N. (2012). Self-assembly of a confined rhodium catalyst for asymmetric hydroformylation of unfunctionalized internal alkenes. J. Am. Chem. Soc. 134, 2860–2863. 10.1021/ja211455j 22280096

[B46] García-Simón C. Gramage-Doria R. Raoufmoghaddam S. Parella T. Costas M. Ribas X.. (2015). Enantioselective hydroformylation by a Rh-catalyst entrapped in a supramolecular metallocage. J. Am. Chem. Soc. 137, 2680–2687. 10.1021/ja512637k 25632976

[B47] Garcia-Viloca M. Gao J. Karplus M. Truhlar D. G. (2004). How enzymes work: analysis by modern rate theory and computer simulations. Science 303, 186–195. 10.1126/science.1088172 14716003

[B48] Gibb C. L. Gibb B. C. (2004). Well-defined, organic nanoenvironments in water: the hydrophobic effect drives a capsular assembly. J. Am. Chem. Soc. 126, 11408–11409. 10.1021/ja0475611 15366865

[B49] Guitet M. Zhang P. Marcelo F. Tugny C. Jiménez-Barbero J. Buriez O. (2013). NHC-capped cyclodextrins (ICyDs): insulated metal complexes, commutable multicoordination sphere, and cavity-dependent catalysis. Angew. Chem. 125, 7354–7359. 10.1002/ange.201301225 23733709

[B50] Gutsche C. D. Muthukrishnan R. (1978). Calixarenes. 1. Analysis of the product mixtures produced by the base-catalyzed condensation of formaldehyde with *para-*substituted phenols. J. Org. Chem. 43, 4905–4906. 10.1021/jo00419a052

[B51] Han X. Xia Q. Huang J. Liu Y. Tan C. Cui Y. (2017). Chiral covalent organic frameworks with high chemical stability for heterogeneous asymmetric catalysis. J. Am. Chem. Soc. 139, 8693–8697. 10.1021/jacs.7b04008 28595384

[B52] Hapiot F. Ponchel A. Tilloy S. Monflier E. (2011). Cyclodextrins and their applications in aqueous-phase metal-catalyzed reactions. Comptes. Rendus. Chim. 14, 149–166. 10.1016/j.crci.2010.04.003

[B53] Hart-Cooper W. M. Clary K. N. Toste F. D. Bergman R. G. Raymond K. N. (2012). Selective monoterpene-like cyclization reactions achieved by water exclusion from reactive intermediates in a supramolecular catalyst. J. Am. Chem. Soc. 134, 17873–17876. 10.1021/ja308254k 23066637

[B54] Hart-Cooper W. M. Zhao C. Triano R. M. Yaghoubi P. Ozores H. L. Burford K. N.. (2015). The effect of host structure on the selectivity and mechanism of supramolecular catalysis of Prins cyclizations. Chem. Sci. 6, 1383–1393. 10.1039/C4SC02735C 29560226 PMC5811099

[B55] Hartmann M. Fischer M. (2012). Amino-functionalized basic catalysts with MIL-101 structure. Microporous Mesoporous Mater. 164, 38–43. 10.1016/j.micromeso.2012.06.044

[B56] Hastings C. J. Pluth M. D. Bergman R. G. Raymond K. N. (2010). Enzymelike catalysis of the Nazarov cyclization by supramolecular encapsulation. J. Am. Chem. Soc. 132, 6938–6940. 10.1021/ja102633e 20443566

[B57] Heinz T. Rudkevich D. M. Rebek J. (1998). Pairwise selection of guests in a cylindrical molecular capsule of nanometre dimensions. Nature 394, 764–766. 10.1038/29501

[B58] Hermes S. Schröter M. K. Schmid R. Khodeir L. Muhler M. Tissler A.. (2005). Metal@MOF: loading of highly porous coordination polymers host lattices by metal organic chemical vapor deposition. Angew. Chem. Int. Ed. 44, 6237–6241. 10.1002/anie.200462515 16130164

[B59] Hong C. M. Morimoto M. Kapustin E. A. Alzakhem N. Bergman R. G. Raymond K. N.. (2018). Deconvoluting the role of charge in a supramolecular catalyst. J. Am. Chem. Soc. 140, 6591–6595. 10.1021/jacs.8b01701 29767972

[B60] Honkala K. Hellman A. Remediakis I. N. Logadottir A. Carlsson A. Dahl S.. (2005). Ammonia synthesis from first-principles calculations. Science 307, 555–558. 10.1126/science.1106435 15681379

[B61] Ishida T. Kinoshita N. Okatsu H. Akita T. Takei T. Haruta M. (2008). Influence of the support and the size of gold clusters on catalytic activity for glucose oxidation. Angew. Chem. Int. Ed. 47, 9265–9268. 10.1002/anie.200802845 18850617

[B62] Jacobs I. van Duin A. C. T. Kleij A. W. Kuil M. Tooke D. M. Spek A. L. (2013). Conformational studies of ligand-template assemblies and the consequences for encapsulation of rhodium complexes and hydroformylation catalysis. Catal. Sci. Technol. 3, 1955–1963. 10.1039/c3cy20665c

[B63] Jans A. C. Gómez-Suárez A. Nolan S. P. Reek J. N. (2016). A switchable gold catalyst by encapsulation in a self-assembled cage. Chem. Eur. J. 22, 14836–14839. 10.1002/chem.201603162 27542162 PMC5053284

[B64] Jiao Y. Tang B. Zhang Y. Xu J. F. Wang Z. Zhang X. (2018). Highly efficient supramolecular catalysis by endowing the reaction intermediate with adaptive reactivity. Angew. Chem. Int. Ed. 57, 6077–6081. 10.1002/anie.201713351 29644773

[B65] Jouffroy M. Gramage-Doria R. Armspach D. Sémeril D. Oberhauser W. Matt D.. (2014). Confining phosphanes derived from cyclodextrins for efficient regio- and enantioselective hydroformylation. Angew. Chem. Int. Ed. 53, 3937–3940. 10.1002/anie.201311291 24590681

[B66] Kaeding W. Chu C. Young L. B. Weinstein B. Butter S. A. (1981). Selective alkylation of toluene with methanol to produce *para*-Xylene. J. Catal. 67, 159–174. 10.1016/0021-9517(81)90269-4

[B67] Kanai J. Kawata N. (1989). Aromatization of n-hexane over galloaluminosilicate and gallosilicate. Appl. Catal. 55, 115–122. 10.1016/S0166-9834(00)82322-2

[B68] Kaphan D. M. Levin M. D. Bergman R. G. Raymond K. N. Toste F. D. (2015). A supramolecular microenvironment strategy for transition metal catalysis. Science 350, 1235–1238. 10.1126/science.aad3087 26785485

[B69] Karpus A. Yesypenko O. Boiko V. Daran J. C. Voitenko Z. Kalchenko V.. (2018). Synthesis of an enantiomerically pure inherently chiral Calix[4]Arene phosphonic acid and its evaluation as an organocatalyst. J. Org. Chem. 83, 1146–1153. 10.1021/acs.joc.7b02312 29323909

[B70] Karroumi J. El Haib E. Manoury E. Benharref A. Daran J-C. Gouygou M. (2015). Selectivity controlled by ligand tuning in the palladium-catalysed cyclocarbonylation: Synthesis of new γ and δ lactones from a natural sesquiterpene. J. Mol. Catal. A Chem. 401, 18–26. 10.1016/j.molcata.2015.02.010

[B71] Koner A. L. Márquez C. Dickman M. H. Nau W. M. (2011). Transition-metal-promoted chemoselective photoreactions at the cucurbituril rim. Angew. Chem. Int. Ed. 50, 545–548. 10.1002/anie.201005317 21069648

[B72] Kubota Y. Takata M. Matsuda R. Kitaura R. Kitagawa S. Kobayashi T. C. (2006). Metastable sorption state of a metal-organic porous material determined by *in situ* synchrotron powder diffraction. Angew. Chem. Int. Ed. 45, 4932–4936. 10.1002/anie.200600976 16807951

[B73] Kuil M. Soltner T. van Leeuwen P. W. Reek J. N. (2006). High-precision catalysts: regioselective hydroformylation of internal alkenes by encapsulated rhodium complexes. J. Am. Chem. Soc. 128, 11344–11345. 10.1021/ja063294i 16939244

[B74] Kuroda Y. Hiroshige T. Ogoshi H. (1990). Epoxidation reaction catalysed by cyclodextrin sandwiched porphyrin in aqueous buffer solution. J. Chem. Soc. Chem. Commun. 22, 1594–1595. 10.1039/c39900001594

[B75] Kuroda Y. Hiroshige T. Sera T. Shiroiwa Y. Tanaka H. Ogoshi H. (1989). Cyclodextrin-sandwiched porphyrin. J. Am. Chem. Soc. 111, 1912–1913. 10.1021/ja00187a073

[B76] Lee E. Y. Jang S. Y. Suh M. P. (2005). Multifunctionality and crystal dynamics of a highly stable, porous metal–organic framework [Zn4O(NTB)2]. J. Am. Chem. Soc. 127, 6374–6381. 10.1021/ja043756x 15853345

[B77] Li S. Y. Xu Y. W. Liu J. M. Su C. Y. (2011). Inherently chiral calixarenes: synthesis, optical resolution, chiral recognition and asymmetric catalysis. Int. J. Mol. Sci. 12, 429–455. 10.3390/ijms12010429 21339996 PMC3039962

[B78] Li Z. Y. Chen Y. Zheng C. Q. Yin Y. Wang L. Sun X. Q. (2017). Highly enantioselective aldol reactions catalyzed by reusable upper rim-functionalized calix[4]arene-based l-proline organocatalyst in aqueous conditions. Tetrahedron 73, 78–85. 10.1016/j.tet.2016.11.052

[B79] Ma D. Li Y. Li Z. (2011). Tuning the moisture stability of metal-organic frameworks by incorporating hydrophobic functional groups at different positions of ligands. Chem. Commun. 47, 7377–7379. 10.1039/c1cc11752a 21625719

[B80] Ma L. Falkowski J. M. Abney C. Lin W. (2010). A series of isoreticular chiral metal-organic frameworks as a tunable platform for asymmetric catalysis. Nat. Chem. 2, 838–846. 10.1038/nchem.738 20861899

[B81] Maksimov A. L. Buchneva T. S. Karakhanov E. A. (2004). Supramolecular calixarene-based catalytic systems in the Wacker-oxidation of higher alkenes. J. Mol. Catal. A Chem. 217, 59–67. 10.1016/j.molcata.2004.03.024

[B82] Márquez F. García H. Palomares E. Fernández L. Corma A. (2001). Spectroscopic evidence in support of the molecular orbital confinement concept: case of anthracene incorporated in zeolites. J. Am. Chem. Soc. 122, 6520–6521. 10.1021/ja0003066

[B83] Matsuda R. Kitaura R. Kitagawa S. Kubota Y. Belosludov R. V. Kobayashi T. C.. (2005). Highly controlled acetylene accommodation in a metal-organic microporous material. Nature 436, 238–241. 10.1038/nature03852 16015325

[B84] Matsuda R. Kitaura R. Kitagawa S. Kubota Y. Kobayashi T. C. Horike S.. (2004). Guest shape-responsive fitting of porous coordination polymer with shrinkable framework. J. Am. Chem. Soc. 126, 14063–14070. 10.1021/ja046925m 15506770

[B85] Mock W. L. Irra T. A. Wepsiec J. P. Adhya M. (1989). Catalysis by cucurbituril. The significance of bound-substrate destabilization for induced triazole formation. J. Org. Chem. 54, 5302–5308. 10.1021/jo00283a024

[B86] Mock W. L. Irra T. A. Wepsiec J. P. Manimaran T. L. (1983). Cycloaddition induced by cucurbituril. A case of Pauling principle catalysis. J. Org. Chem. 48, 3619–3620. 10.1021/jo00168a070

[B87] Monnereau L. Sémeril D. Matt D. (2011). High efficiency of cavity-based triaryl-phosphines in nickel-catalysed Kumada-Tamao-Corriu cross-coupling. Chem. Commun. 47, 6626–6628. 10.1039/c0cc05805j 21544285

[B88] Monnereau L. Semeril D. Matt D. (2012). A supramolecular variant of the Suzuki-Miyaura reaction. Actual. Chim. 359, 8–12. 10.1049/c0cc05805j

[B89] Monnereau L. Sémeril D. Matt D. (2013). Calixarene-derived mono-iminophosphoranes: highly efficient ligands for palladium- and nickel-catalysed cross-coupling. Adv. Synth. Catal. 355, 1351–1360. 10.1002/adsc.201300091

[B90] Monnereau L. Sémeril D. Matt D. Gourlaouen C. (2017). Catalytic behaviour of calixarenylphosphanes in nickel-catalysed suzuki–miyaura cross-coupling. Eur. J. Inorg. Chem. 18, 581–586. 10.1002/ejic.201601351

[B91] Müller U. Luinstra G. Yaghi O. M. (2003). Process for Producing Polyalkylene Carbonates. Germany: Basf Aktiengesellschaft. Patent No WO/2004/037895 (Mannheim).

[B92] Nishioka Y. Yamaguchi T. Kawano M. Fujita M. (2008). Asymmetric [2 + 2] olefin cross photoaddition in a self-assembled host with remote chiral auxiliaries. J. Am. Chem. Soc. 130, 8160–8161. 10.1021/ja802818t 18540605

[B93] Ogawa H. Koh T. Taya K. Chihara T. (1994). Catalysis at the toluene/water interface: octadecyl immobilized H-ZSM-5 Catalyst promoted hydrolysis of water-insoluble esters. J. Catal. 148, 493–500. 10.1006/jcat.1994.1235

[B94] Opelt S. Krug V. Sonntag J. Hunger M. Klemm E. (2012). Investigations on stability and reusability of [Pd(2-pymo)(2)](n) as hydrogenation catalyst. Microporous Mesoporous Mater. 147, 327–333. 10.1016/j.micromeso.2011.07.003

[B95] Opelt S. Türk S. Dietzsch E. Henschel A. Kaskel S. Klemm E. (2008). Preparation of palladium supported on MOF-5 and its use as hydrogenation catalyst. Catal. Commun. 9, 1286–1290. 10.1016/j.catcom.2007.11.019

[B96] Otte M. Kuijpers P. F. Troeppner O. Ivanović-Burmazović I. Reek J. N. de Bruin B. (2013). Encapsulation of metalloporphyrins in a self-assembled cubic M8L6 cage: a new molecular flask for cobalt-porphyrin-catalysed radical-type reactions. Chemistry 19, 10170–10178. 10.1002/chem.201301411 23821458

[B97] Otte M. Kuijpers P. F. Troeppner O. Ivanović-Burmazović I. Reek J. N. de Bruin B. (2014). Encapsulated cobalt-porphyrin as a catalyst for size-selective radical-type cyclopropanation reactions. Chemistry 20, 4880–4884. 10.1002/chem.201400055 24664657

[B98] Paillaud J. L. Harbuzaru B. Patarin J. Bats N. (2004). Extra-large-pore zeolites with two-dimensional channels formed by 14 and 12 rings. Science 304, 990–992. 10.1126/science.1098242 15143276

[B99] Pompliano D. L. Peyman A. Knowles J. R. (1990). Stabilization of a reaction intermediate as a catalytic device: definition of the functional role of the flexible loop in triosephosphate isomerase. Biochemistry 29, 3186–3194. 10.1021/bi00465a005 2185832

[B100] Praneeth V. K. K. Ringenberg M. R. Ward T. R. (2012). Redox-active ligands in catalysis. Angew. Chem. Int. Ed. 51, 10228–10234. 10.1002/anie.201204100 22996755

[B101] Raynal M. Ballester P. Vidal-Ferran A. van Leeuwen P. W. N. M. (2014). Supramolecular catalysis. Part 2: artificial enzyme mimics. Chem. Soc. Rev. 43, 1734–1787. 10.1039/c3cs60037h 24365792

[B102] Sahin O. Eymur S. Uyanik A. Akceylan E. Yilmaz M. (2018). Chiral Calix[4]arenes-bearing prolinamide functionality as organocatalyst for asymmetric direct aldol reactions in water. Polycycl. Aromat. Compd. 38, 168–179. 10.1080/10406638.2016.1176058

[B103] Schröder F. Esken D. Cokoja M. van den Berg M. W. Lebedev O. I. Van Tendeloo G.. (2008). Ruthenium nanoparticles inside porous [Zn4O(bdc)3] by Hydrogenolysis of adsorbed [Ru(cod)(cot)]: a solid-state reference system for surfactant-stabilized ruthenium colloids. J. Am. Chem. Soc. 130, 6119–6130. 10.1021/ja078231u 18402452

[B104] Slagt V. F. Kamer P. C. van Leeuwen P. W. Reek J. N. (2004). Encapsulation of transition metal catalysts by ligand-template directed assembly. J. Am. Chem. Soc. 126, 1526–1536. 10.1021/ja0386795 14759211

[B105] Slagt V. F. Reek J. N. H. Kamer P. C. J. van Leeuwen P. W. N. M. (2001). Assembly of encapsulated transition metal catalysts. Angew. Chem. 113, 4401–4404. 10.1002/1521-3773(20011119)40:22<4271::AID-ANIE4271>3.0.CO;2-H 29712091

[B106] Steitz T. A. Harrison R. Weber I. T. Leahy M. (2008). Ligand-Induced Conformational Changes in Proteins Ciba Foundation Symposium 93 - Mobility and Function, in Proteins and Nucleic Acids (KGaA, Weinheim: WILEY-VCH Verlag GmbH & Co.), 25–46. 10.1002/9780470720752.ch3 6301781

[B107] Trost B. M. van Vranken D. L. Bingel C. (1992). A Modular approach for ligand design for asymmetric allylic alkylations via enantioselective palladium-catalyzed ionizations. J. Am. Chem. Soc. 114, 9327–9343. 10.1021/ja00050a013

[B108] Ueda Y. Ito H. Fujita D. Fujita M. (2017). Permeable self-assembled molecular containers for catalyst isolation enabling two-step cascade reactions. J. Am. Chem. Soc. 139, 6090–6093. 10.1021/jacs.7b02745 28402111

[B109] Uemura T. Hiramatsu D. Kubota Y. Takata M. Kitagawa S. (2007). Topotactic linear radical polymerization of divinylbenzenes in porous coordination polymers. Angew. Chem. Int. Ed. 46, 4987–4990. 10.1002/anie.200700242 17514689

[B110] Uemura T. Kitaura R. Ohta Y. Nagaoka M. Kitagawa S. (2006). Nanochannel-promoted polymerization of substituted acetylenes in porous coordination polymers. Angew. Chem. Int. Ed. 45, 4112–4116. 10.1002/anie.200600333 16721889

[B111] van Leeuwen P. W. N. M. (2004). Homogeneous Catalysis: Understanding the Art. Netherlands: Springer.

[B112] Vermoortele F. Vandichel M. Van de Voorde B. Ameloot R. Waroquier M. Van Speybroeck V.. (2012). Electronic effects of linker substitution on Lewis acid catalysis with metal-organic frameworks. Angew. Chem. Int. Ed. 51, 4887–4890. 10.1002/anie.201108565 22488675

[B113] Vriezema D. M. Comellas Aragonès M. Elemans J. A. Cornelissen J. J. Rowan A. E. Nolte R. J. (2005). Self-assembled nanoreactors. Chem. Rev. 105, 1445–1489. 10.1021/cr0300688 15826017

[B114] Waller P. J. Gándara F. Yaghi O. M. (2015). Chemistry of covalent organic frameworks. Acc. Chem. Res. 48, 3053–3063. 10.1021/acs.accounts.5b00369 26580002

[B115] Wang Q. Q. Gonell S. Leenders S. H. Dürr M. Ivanović-Burmazovi,ć I. Reek J. N. (2016). Self-assembled nanospheres with multiple endohedral binding sites pre-organize catalysts and substrates for highly efficient reactions. Nat. Chem. 8, 225–230. 10.1038/nchem.2425 26892553

[B116] Wang Z. J. Clary K. N. Bergman R. G. Raymond K. N. Toste F. D. (2013). A supramolecular approach to combining enzymatic and transition metal catalysis. Nat. Chem. 5, 100–103. 10.1038/nchem.1531 23344446

[B117] Wu P. Wang J. Li Y. He C. Xie Z. Duan C. (2011). Luminescent sensing and catalytic performances of a multifunctional lanthanide-organic framework comprising a triphenylamine moiety. Adv. Funct. Mater. 21, 2788–2794. 10.1002/adfm.201100115

[B118] Yamaguchi T. Fujita M. (2008). Highly selective photomediated 1,4-radical addition to o-quinones controlled by a self-assembled cage. Angew. Chem. Int. Ed. 47, 2067–2069. 10.1002/anie.200705139 18247442

[B119] Yoshizawa M. Tamura M. Fujita M. (2006). Diels-alder in aqueous molecular hosts: unusual regioselectivity and efficient catalysis. Science 312, 251–254. 10.1126/science.1124985 16614218

[B120] Yu F. Poole D. Mathew S. Yan N. Hessels J. Orth N.. (2018). Control over electrochemical water oxidation catalysis by preorganization of molecular ruthenium catalysts in self-assembled nanospheres. Angew. Chem. Int. Ed. 57, 11247–11251. 10.1002/anie.201805244 29975448 PMC6120458

[B121] Zhang J. Han X. Wu X. Liu Y. Cui Y. (2017a). Multivariate chiral covalent organic frameworks with controlled crystallinity and stability for asymmetric catalysis. J. Am. Chem. Soc. 139, 8277–8285. 10.1021/jacs.7b03352 28537721

[B122] Zhang J. Wang L. Shao Y. Wang Y. Gates B. C. Xiao F. S. (2017b). A Pd@Zeolite catalyst for nitroarene hydrogenation with high product selectivity by sterically controlled adsorption in the zeolite micropores. Angew. Chem. Int. Ed. 56, 9747–9751. 10.1002/anie.201703938 28503914

[B123] Zheng L. Sonzini S. Ambarwati M. Rosta E. Scherman O. A. Herrmann A. (2015). Turning Cucurbit[8]uril into a supramolecular nanoreactor for asymmetric catalysis. Angew. Chem. Int. Ed. 54, 13007–13011. 10.1002/anie.201505628 26383272 PMC4643185

